# Design and evaluation of tadpole-like conformational antimicrobial peptides

**DOI:** 10.1038/s42003-023-05560-0

**Published:** 2023-11-18

**Authors:** Ziyi Tang, Wuqiao Jiang, Shuangli Li, Xue Huang, Yi Yang, Xiaorong Chen, Jingyi Qiu, Chuyu Xiao, Ying Xie, Xu Zhang, Jianguo Li, Chandra Shekhar Verma, Yun He, Aimin Yang

**Affiliations:** 1 Chongqing Institute of Green and Intelligent Technology, Chinese Academy of Sciences, Chongqing, 400714 China; 2 School of Pharmaceutical Sciences, Chongqing University, Chongqing, 401331 China; 3 School of Life Sciences, Chongqing University, Chongqing, 401331 China; 4 National Centre for Magnetic Resonance in Wuhan, State Key Laboratory of Magnetic Resonance and Atomic and Molecular Physics, Innovation Academy for Precision Measurement Science and Technology, Chinese Academy of Sciences, Wuhan, 430071 China; 5 Bioinformatics Institute, A∗STAR, 30 Biopolis Street, Matrix, Singapore, 138671 Singapore; 6 Singapore Eye Research Institute, Singapore, 169856 Singapore; 7 Department of Biological Sciences, National University of, Singapore, 117543 Singapore; 8 School of Biological Sciences, Nanyang Technological University, Singapore, 637551 Singapore; 9 BayRay Innovation Center, Shenzhen Bay Laboratory, Shenzhen, 518132 China

**Keywords:** Peptides, Antibiotics

## Abstract

Antimicrobial peptides are promising alternatives to conventional antibiotics. Herein, we report a class of “tadpole-like” peptides consisting of an amphipathic α-helical head and an aromatic tail. A structure-activity relationship (SAR) study of “tadpole-like” temporin-SHf and its analogs revealed that increasing the number of aromatic residues in the tail, introducing Arg to the α-helical head and rearranging the peptide topology dramatically increased antimicrobial activity. Through progressive structural optimization, we obtained two peptides, HT2 and RI-HT2, which exhibited potent antimicrobial activity, no hemolytic activity and cytotoxicity, and no propensity to induce resistance. NMR and molecular dynamics simulations revealed that both peptides indeed adopted “tadpole-like” conformations. Fluorescence experiments and electron microscopy confirmed the membrane targeting mechanisms of the peptides. Our studies not only lead to the discovery of a series of ultrashort peptides with potent broad-spectrum antimicrobial activities, but also provide a new strategy for rational design of novel “tadpole-like” antimicrobial peptides.

## Abstract (web summary)

Here, through progressive structural optimization, two “tadpole-like” antimicrobial peptides are constructed with an amphipathic α-helical head and an aromatic tail activity. These exhibit potent antimicrobial activity, no hemolytic activity and cytotoxicity, and no propensity to induce resistance.

## Introduction

Antimicrobial resistance has emerged as an imminent threat to global public health due to the excessive use and misuse of antibiotics^1,2^. Without the intervention of national policies restricting the use of antibiotics, ten million deaths are predicted from antimicrobial-resistant infections each year by 2050^3,4^. In the meantime, the traditional antibiotic pipeline has almost been exhausted. Therefore, there is an urgent need for alternative strategies to resolve the crisis of antimicrobial resistant infections^2,5–7^. One promising strategy to tackle this global threat is to use antimicrobial peptides (AMPs), which have advantageous low probability of resistance development because of their direct action on bacterial membranes^8,9^.

AMPs are a class of short peptides ubiquitously found in all living forms, which play a crucial role in the natural innate immune system^10–13^. Unlike conventional antibiotics that generally target metabolic enzymes, many AMPs can rapidly disrupt bacterial membranes, thereby resulting in cell damage and death of microorganisms^14,15^. The action mechanism of AMPs suggests that AMPs may be less susceptible to resistance, enabling AMPs with great potential as alternatives to conventional antibiotics. However, most natural AMPs have severe drawbacks as potential antibiotics, such as low or moderate antimicrobial activity, high toxicity, and relatively high molecular weight with more than 20 amino acid residues, preventing them from being developed into therapeutics^16–18^. To overcome these limitations, the rational design of synthetic ultrashort peptides based on natural templates promises to accelerate the generation of novel lead antimicrobial compounds.

Due to their relatively small size and synthetic accessibility, amphipathic α-helical AMPs have been extensively studied, and a substantial amount of structure-activity relationship (SAR) data is available for rational optimization of AMPs^10,13^. Aromatic residues appear to play special roles in mediating peptide-membrane interactions because of their interface-seeking properties^19–22^. Poly-aromatic residues also have been shown to stabilize peptide structure and to permeabilize bacterial membranes^23–27^. Given the effective antimicrobial activity of amphipathic α-helical AMPs and the importance of clustered aromatic residues in antimicrobial peptides, we presume that a combination of an amphipathic α-helical segment and a polyaromatic tail may enhance antimicrobial activity. In order to verify this hypothesis, we propose a class of AMPs displaying a “tadpole-like” conformation consisting of an amphipathic α-helical head and an aromatic tail.

In the present study, temporin-SHf, the smallest natural amphibian antimicrobial peptide consisting of eight residues characterized from the skin of the North African frog *Pelophylax saharicus*^28^, was selected as a starting peptide to validate the concept of “tadpole-like” antimicrobial peptides. Temporin-SHf adopts a well-defined amphipathic α-helical structure and a disordered dual-Phe motif that is arranged in a tail-to-head fashion^28^, which meets our desired “tadpole-like” conformation. Natural temporin-SHf peptide displays weak antimicrobial activity. Taking this peptide as a hit compound, we performed a detailed structure-activity relationship study on how the “tadpole-like” conformation modulates antimicrobial activity. Through structural optimization, we present a model in which the unique “tadpole-like” antimicrobial peptide interacts with a bacterial membrane and displays promising antimicrobial activity. Moreover, we obtained a series of ultrashort peptide analogs which displayed potent activities against clinical Gram-positive and Gram-negative multidrug-resistant bacterial strains.

## Results

### Peptide design principles based on “tadpole-like” conformation

**Fig. 1:**
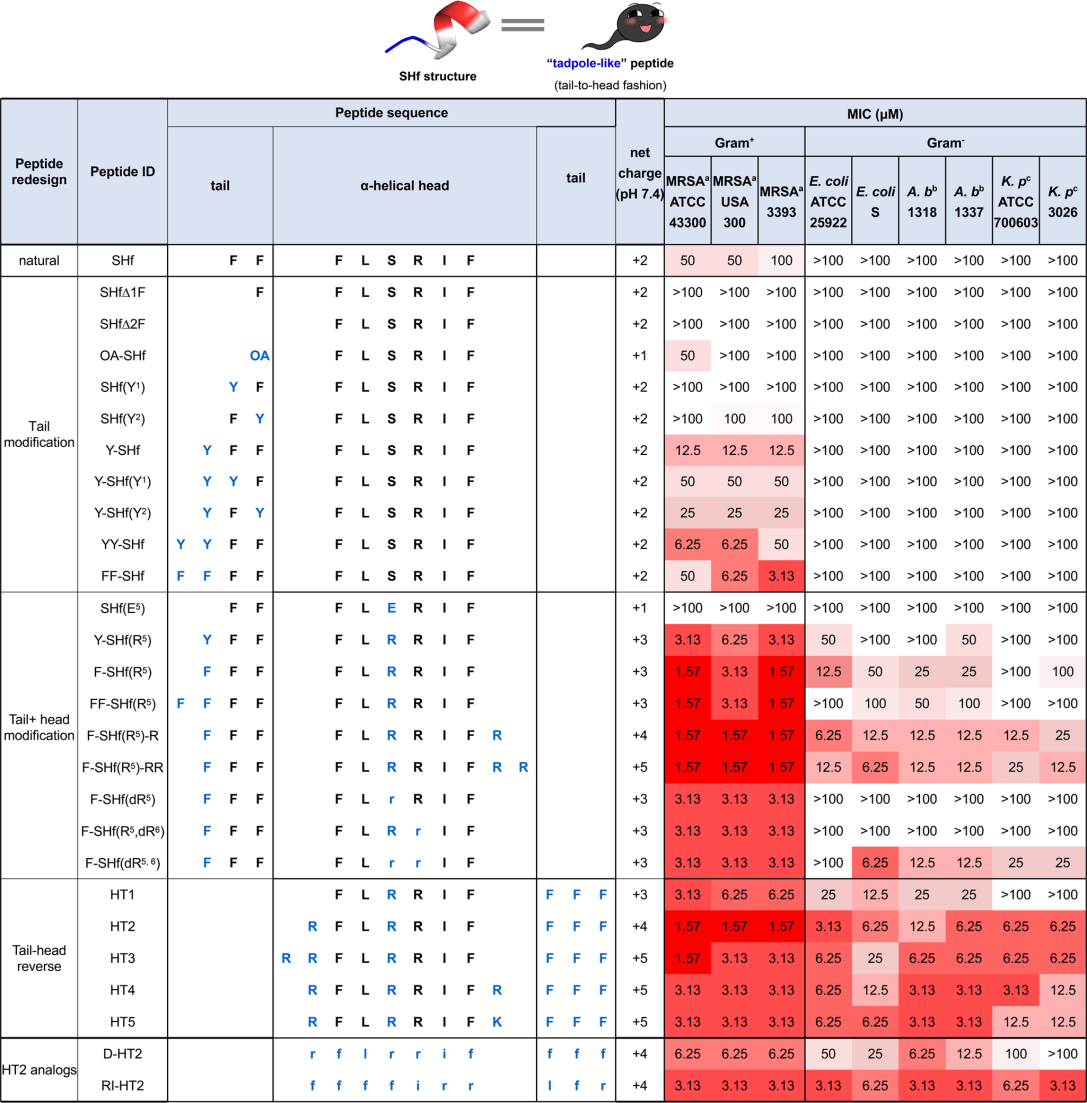
Temporin-SHf analogs design. Temporin-SHf shows “tadpole-like” conformation consisting of a well-defined weak amphipathic α-helical structure and a disordered two-Phe tail motif arranging in a tail-to-head fashion. To explore the structure-activity relationship (SAR) of the “tadpole-like” antimicrobial peptides, the hydrophobic aromatic tail, α-helical head and their arrangement order were redesigned. In peptide sequences, uppercase letters indicate L-amino acids, whereas the lowercase letters indicate D-amino acids. Mutated residues are colored in blue. MRSA methicillin-resistant *Staphylococcus aureus*; *A. b*., *Acinetobacter baumannii*; *K. p*., *Klebsiella pneumonia*.

Temporin-SHf adopts a well-defined amphipathic α-helical structure from the residue Phe3 to Phe8, whereas the N-terminal Phe1 and Phe2 residues are disordered in the presence of membrane mimics^28^. The aromatic N-terminus and the α-helical segment are arranged in a tail-to-head fashion. Our studies showed that temporin-SHf exhibited weak antimicrobial activity against Gram-positive bacteria and no detectable activity against Gram-negative bacteria at the concentration range tested (1–100 μM) (Fig. 1), which is consistent with previous reports^28,29^. To gain in-depth knowledge of the effect of “tadpole-like” conformations on antimicrobial activity, we designed a series of peptide analogs by taking into account net charge, amphipathicity of α-helical structure, hydrophobicity of N-terminal tail, topological rearrangement of α-helical segment and N-terminal tail, as well as partial or complete D-amino acid substitution (Fig. 1). All peptide analogs were synthesized by employing a 9-fluorenylmethoxycarbonyl (Fmoc)-based solid phase peptide synthesis strategy. The measured molecular weights and purities of the peptides were confirmed by mass spectrometry and reverse phase high-performance liquid chromatography (RP-HPLC) analyses, respectively (Table S1). The minimum inhibitory concentrations (MICs) of all peptide analogs against a wide range of Gram-negative and Gram-positive bacterial strains were determined.

### Structure-activity relationship of the N-terminal tail of “tadpole-like” conformation

Natural temporin-SHf has weak antimicrobial activity against *Staphylococcus aureus* and methicillin-resistant *Staphylococcus aureus* (MRSA) with MICs of 50–100 µM (Fig. 1). We first examined the role of the aromatic N-terminus in the antimicrobial activity of temporin-SHf. To such a purpose, the aromatic N-terminus was partially or completely deleted to obtain analogs SHf∆1 F and SHf∆2 F, respectively, or replaced by a hydrophobic octanoic acid (OA) chain to give the analog OA-SHf. Furthermore, Phe1 and Phe2 residues were substituted by Tyr (Fig. 1). We found that the deletion of N-terminal Phe residues resulted in loss of the antimicrobial activity at peptide concentration of 100 µM. Similarly, antimicrobial activity was not detected when the Phe residue was replaced by the hydrophobic octanoic acid (OA) chain or Tyr. In addition, we examined whether adding aromatic residues (Phe or Tyr) to the N-terminus affects antimicrobial activity. Interestingly, two analogs, YY-SHf and FF-SHf showed potent antimicrobial activity against three MRSA strains with MICs of 3.13–50 µM. Therefore, the aromatic N-terminus is required for the antimicrobial activity of temporin-SHf, and adding aromatic residues to the N-terminus appear to increase antimicrobial activity.

### Structure-activity relationship of α-helical head of “tadpole-like” conformation

The α-helical structure of temporin-SHf consists of six amino acid residues. The helical wheel projection showed that only two polar residues, Ser5 and Arg6, were separated on the hydrophilic surface of the helical cylinder (Fig. S1a). Hence, to enhance the amphipathicity of the α-helical head, Ser5 was substituted by a residue with a higher polarity-index, such as negatively charged Glu or positively charged Arg. The helical wheel projection showed that one or two basic residues adding to the C-terminus are located on the hydrophilic surface of the helical cylinder (Fig. S1b). Furthermore, it has been reported that increasing the net positive charge leads to better antimicrobial activity^11,13,30^. Thus, one or two basic Arg residues were added to the C-terminus to give analogs F-SHf(R^5^)-R and F-SHf(R^5^)-RR, respectively. In addition, to explore the effect of chirality of basic residues on the antimicrobial activity, D-Arg was used to substitute L-Arg at position 5 or/and position 6 (Fig. 1).

The results showed that the replacement of Ser5 by the hydrophilic residue Glu led to a decrease in the antimicrobial activity. Since bacterial membranes are negatively charged, it seems reasonable that the introduction of an acidic residue is detrimental to the activity. On the other hand, the substitution with Arg at position 5 greatly increased the antimicrobial activity against MRSA. In particular, FF-SHf(R^5^) showed very good antimicrobial activity against MRSA ATCC 43300 and 3393 with MICs of 1.57 µM. Moreover, F-SHf(R^5^)-R and F-SHf(R^5^)-RR also displayed strong antimicrobial activity against MRSA ATCC 43300 and 3393 with MICs of 1.57 µM. Interestingly, both peptides F-SHf(R^5^)-R and F-SHf(R^5^)-RR exhibited decent antimicrobial activity against the tested Gram-negative bacterial strains with MICs of 6.25–25 µM, whereas natural temporin-SHf had no detectable antimicrobial activity at concentrations up to 100 µM. In addition, the substitution with D-Arg at position 5/6 resulted in some enhancement to the antimicrobial activity of F-SHf against Gram-negative bacterial strains.

Therefore, our findings suggested that the substitution with Arg at position 5 together with the addition of Arg residues to the C-terminus enhances antimicrobial activity.

### Effect of topological rearrangement on antimicrobial activity

Temporin-SHf peptide is arranged in a unique hydrophobic tail-to-α-helical head fashion. We therefore explored the effect of conformational topology of temporin-SHf on antimicrobial activity. Topological rearrangement of F-SHf(R^5^) was carried out using circular permutation to generate a new peptide, termed as HT1, in a head-to-tail fashion (Fig. 1). Starting from this new peptide, we also examined the effect of increasing the net positive charge and α-helical amphipathicity on the antimicrobial activity in this fashion by taking into account our proposed structure-activity relationships of the N-terminal tail and α-helical head of “tadpole-like” conformation.

Peptide HT1, originating from reversing tail-to-head fashion of F-SHf(R^5^) to head-to-tail fashion, displayed interesting antimicrobial activity against MRSA with MICs of 3.13-6.25 µM and against Gram-negative *Acinetobacter baumannii* with MIC of 25 µM (Fig. 1). Based on the above structure-activity relationship of the α-helical head, we also examined the effect on the antimicrobial activity of HT1 by adding one or two Arg residues to the helical head. The resulting peptides, HT2 and HT3, exhibited comparable antimicrobial activity against Gram-positive bacterial strains with MICs of 1.57-3.13 µM. More importantly, both peptides HT2 and HT3 showed very promising antimicrobial activity against Gram-negative bacterial strains, such as *A. baumannii* and *Klebsiella pneumoniae*, with MICs of 6.25 µM. In addition, the helical wheel projection showed that one additional Arg or Lys placed in the region between the α-helical head and the hydrophobic aromatic tail was located on the hydrophilic surface of the helical cylinder (Fig. S1c). The resulting peptides HT4 and HT5, containing very polar residue Arg or Lys at the C-terminus of the helical head respectively, displayed even stronger antimicrobial activity against all tested bacterial strains with MICs of 3.13–12.5 µM (Fig. 1). Thus, topological rearrangement of F-SHf(R^5^) using circular permutation greatly enhanced antimicrobial activity.

Retro-inverso peptides possess reversed sequences and chirality compared to the parent molecules and meanwhile maintain identical sidechain conformations compared to the parent peptide^31,32^. We therefore explored the effect of reversed sequences and chirality of temporin-SHf analogs on antimicrobial activity. HT2 was selected as a starting peptide because of its potent antimicrobial activity. In this regard, we synthesized two HT2 derivatives, including D-HT2 by substituting the L-amino acid residues with D-amino acids and retro-inverso HT2 (RI-HT2) consisting of D-amino acid residues in a reversed sequence. We found that RI-HT2, not D-HT2, showed comparable antimicrobial activity to HT2 against all tested Gram-positive and Gram-negative bacterial strains (Fig. 1).

Through our optimization, several new “tadpole-like” peptides, including F-SHf(R^5^), F-SHf(R^5^)-R, F-SHf(R^5^)-RR, HT1-5 and RI-HT2, displayed stronger and broader spectrum activity against both Gram-positive and Gram-negative bacterial strains than the parent peptide temporin-SHf and other analogs.

### Hemolytic activity, cytotoxicity and stability

**Fig. 2:**
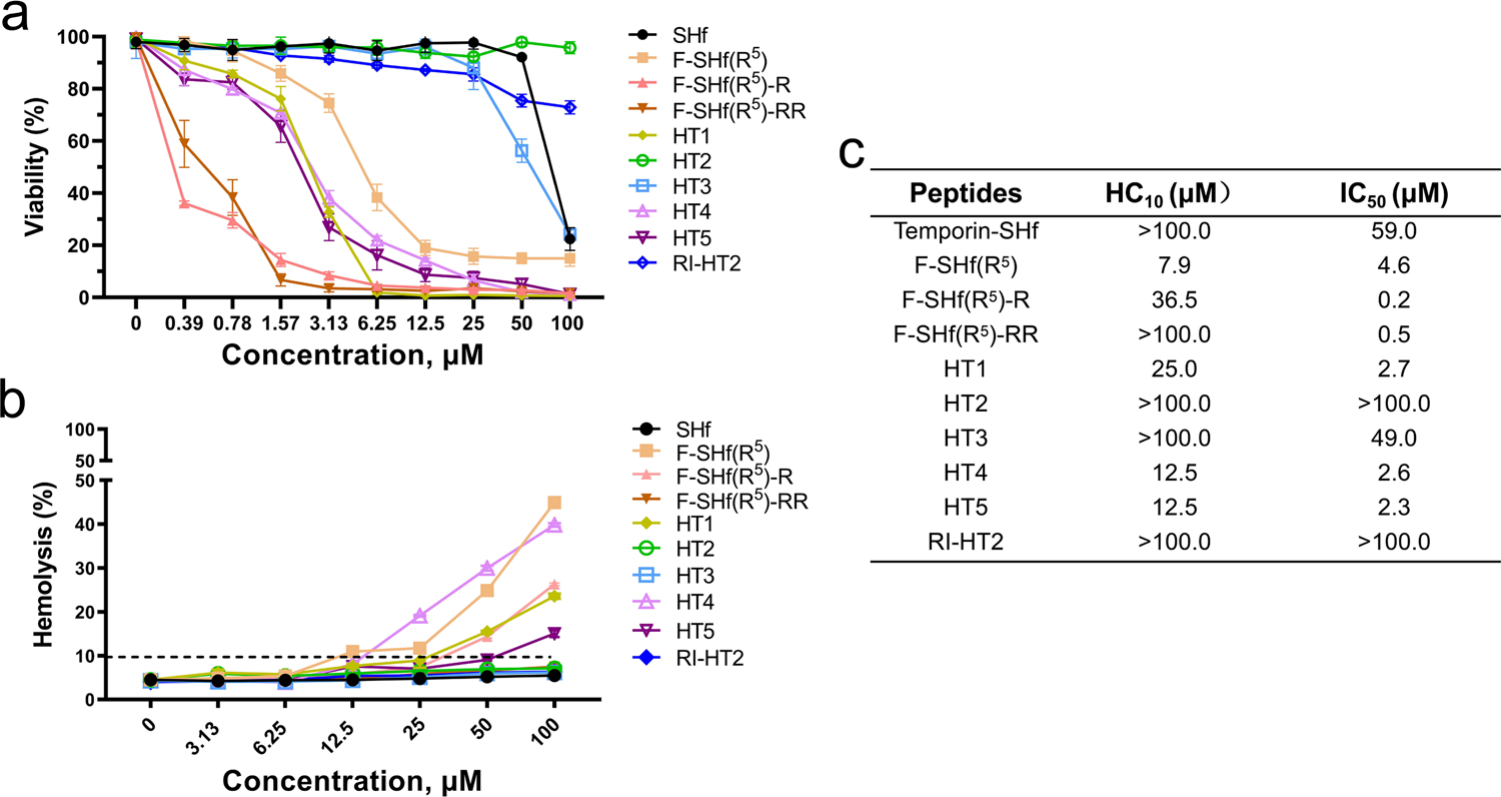
Hemolysis and cytotoxicity of SHf and its analogs. **a** Cytotoxicity of native peptide SHf and its analogs in HEK293T mammalian cell. Toxicity values are the mean of *n* = 3. **b** Hemolysis of native peptide SHf and its analogs. Hemolysis values are the mode of 6 independent biological replicates. **c** Data presentation of (**a**, **b**). Mammalian cell cytotoxicity was displayed as IC_50_, and red blood cell hemolysis as HC_10_ (concentration inducing 10% hemolysis).

We next evaluated the effect of various amino acid mutations and topological rearrangements on peptide hemolytic activity and cytotoxicity. Natural temporin-SHf peptide did not present hemolytic activity in sheep red blood cell assay at the concentration range tested (3.13–100 μM), but it was toxic (IC_50_ was 59.0 μM) against the human embryonic kidney cells (HEK293T) (Fig. 2). Similarly, several peptides, including F-SHf(R^5^), F-SHf(R^5^)-R, F-SHf(R^5^)-RR, HT1, HT4 and HT5, induced slight hemolytic activity and showed high toxicity against HEK293T cells. The IC_50_ of these peptides are close to their MICs, suggesting low biocompatibility for these peptides. In addition, HT3 is similar to temporin-SHf in terms of cellular toxicity and hemolysis. Interestingly, HT2 and RI-HT2 did not present hemolytic activity against sheep red blood cells and cytotoxicity against HEK293T cells at the concentration range tested (Fig. 2).

We next evaluated the stability of HT2 and RI-HT2 by testing their antimicrobial activity against MRSA USA 300 and *K. pneumoniae* ATCC 700603 exposed to the high concentration of salt, low pH and serum. We found that a high concentration of salt (150 mM NaCl) and low pH (5.5 or 6.4) caused a doubled MIC value of both peptides against *K. pneumoniae*, but not MRSA (Table 1). In the case of serum, HT2 exhibited no change of the MIC value against *K. pneumoniae* and MRSA. Similar with HT2, no change of MIC of RI-HT2 was observed in 10% and 20% fetal bovine serum (FBS) against MRSA, whereas the same treatment severely attenuated the activity of RI-HT2 against *K. pneumoniae* (Table 1). These results suggested that HT2 and RI-HT2 show tolerable stability exposed to salt, low pH and serum.

**Table 1:** MIC Values (µM) of peptides against MRSA USA 300 and *K. pneumoniae* ATCC 700603 in the presence of fetal bovine serum (FBS), salt, and pH.

peptides	Normal medium	pH 5.5	pH 6.4	150 mM NaCl	10% FBS	20% FBS
MIC against MRSA USA 300 (µM)						
HT2	1.56	3.13	3.13	3.13	1.56	1.56
RI-HT2	3.13	3.13	3.13	3.13	3.13	3.13
MIC against *K. pneumoniae* ATCC 700603 (µM)						
HT2	6.25	12.5	6.25	12.5	6.25	6.25
RI-HT2	6.25	12.5	12.5	12.5	>100	>100

Collectively, our results suggested that HT2 and RI-HT2 consisting of 10-amino acid residues have the highest selectivity towards Gram-positive and Gram-negative bacteria, and excellent biocompatibility towards mammalian cells as well as good stability.

### Time-kill analysis

**Fig. 3:**
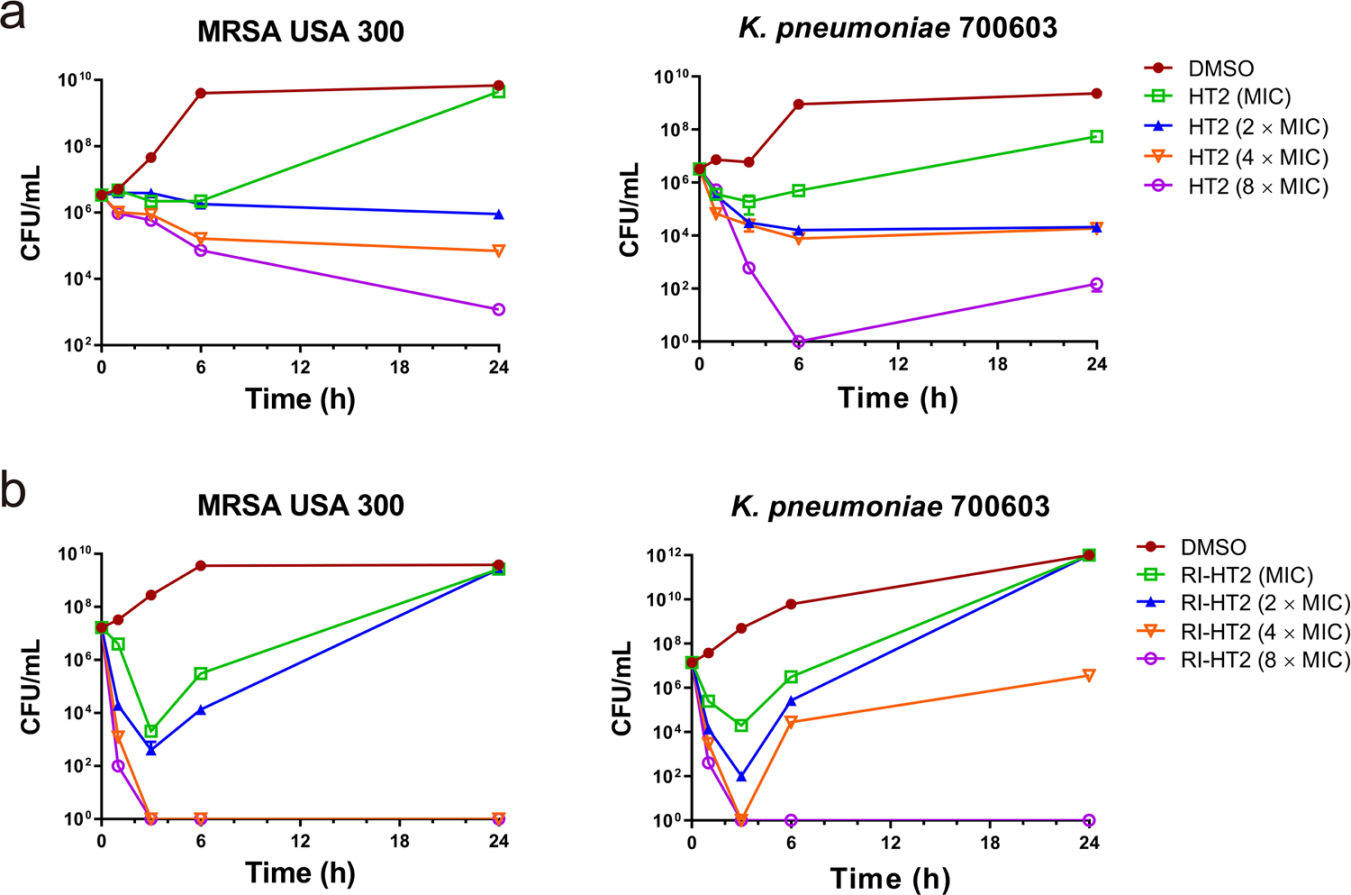
Time-kill assays of HT2 and RI-HT2. **a** Time-kill assays of HT2. The left panel showed the time-kill kinetics of HT2 against MRSA USA 300, and the right panel showed the time-kill kinetics of HT2 against *K. pneumoniae* ATCC 700603. Data are mean ± SD for n = 3 biologically independent samples. **b** Time-kill assays of RI-HT2. The left panel showed the time-kill kinetics of RI-HT2 against MRSA USA 300, and the right panel showed the time-kill kinetics of RI-HT2 against *K. pneumoniae* ATCC 700603. Data are mean ± SD for *n* = 3 biologically independent samples.

To further evaluate the antimicrobial effect of HT2 and RI-HT2, we performed time killing experiments for a panel of bacteria, including Gram-positive strains *S. aureus* ATCC 25923 or MRSA USA 300 and a Gram-negative strain *K. pneumoniae* ATCC 700603. The time-kill curves of HT2 against MRSA USA 300 and *K. pneumoniae* ATCC 700603 are similar, and in a concentration-dependent manner (Fig. 3a). HT2 with a higher concentration (16 × MIC) completely inhibited bacterial growth of *S. aureus* ATCC 25923 in 1 h (Fig. S2). MRSA USA 300 and *K. pneumoniae* ATCC 700603 cannot completely be killed by treatment with HT2 at 8 × MIC after 24 h (Fig. 3a), while RI-HT2 had rapid bactericidal activity against MRSA USA 300 and *K. pneumoniae* ATCC 700603 (Fig. 3b). Specifically, *K. pneumoniae* ATCC 700603 was completely killed in 3 h at 8 × MIC of RI-HT2 while the same inhibitory effect on MRSA USA 300 was achieved at 4 × MIC of RI-HT2. Overall, the rapid killing kinetics of HT2 and RI-HT2 against Gram-positive and Gram-negative bacteria implies that HT2 and RI-HT2 disrupt the integrity of bacterial cell membranes^33^.

### Effect of peptides on membrane integrity

The natural peptide temporin-SHf exerts its antibacterial effect by disrupting the acyl chain packing of the anionic lipid bilayer, and by disintegrating the membrane probably via the carpet mechanism^28^. In order to gain insights into the mechanisms of action by which these peptides kill bacterial cells, we assessed whether peptides are capable of depolarizing the cytoplasmic membrane and permeabilizing the membrane of bacteria.

**Fig. 4:**
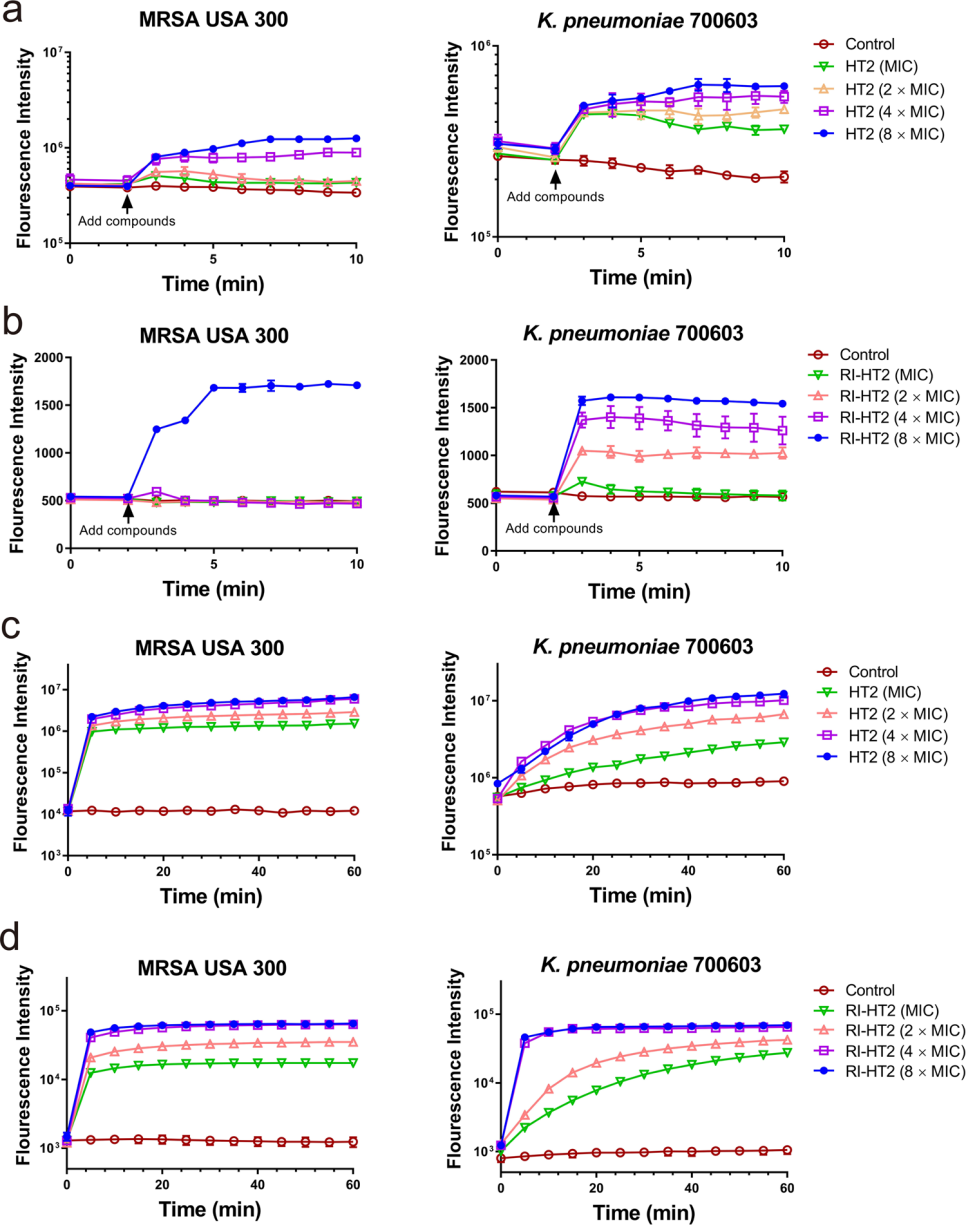
HT2 and RI-HT2 showed depolarization and permeability of cytoplasmic membrane. **a** DiSC_3_(5) test of HT2 against MRSA USA 300 (left panel) and *K. pneumoniae* ATCC 700603 (right panel). **b** DiSC_3_(5) test of RI-HT2 against MRSA USA 300 (left panel) and *K. pneumoniae* ATCC 700603 (right panel). **c** SYTOX Green assay of HT2 against MRSA USA 300 (left panel) and *K. pneumoniae* ATCC 700603 (right panel). **c** SYTOX Green assay of HT2 against MRSA USA 300 (left panel) and *K. pneumoniae* ATCC 700603 (right panel). **d** SYTOX Green assay of RI-HT2 against MRSA USA 300 (left panel) and *K. pneumoniae* ATCC 700603 (right panel). Each data point is the mean of *n* = 3.

Permeabilization activity against membranes of MRSA and *K. pneumoniae* were investigated using the DiSC_3_(5) uptake assay^34^. In MRSA USA 300, at concentrations equivalent to 4 × MIC of HT2 and 8 × MIC of RI-HT2, the fluorescence intensity was found to have changed significantly (Fig. 4a, b). The fluorescence intensity increased upon treatment with HT2 and RI-HT2 in *K. pneumoniae* ATCC 700603 in a time- and dose-dependent manner (Fig. 4a, b). To further confirm membrane-targeting activity of the peptides, a DNA-binding dye SYTOX Green was used as a reporter to evaluate membrane permeabilization^35,36^. In MRSA USA 300, different concentrations of HT2 and RI-HT2 reached the maximum fluorescence intensity within 10 and 15 min (Fig. 4c, d). In *K. pneumoniae* ATCC 700603, the fluorescence signal reached the highest intensity within 40 minutes, and the effect was dose-dependent (Fig. 4c, d).

**Fig. 5:**
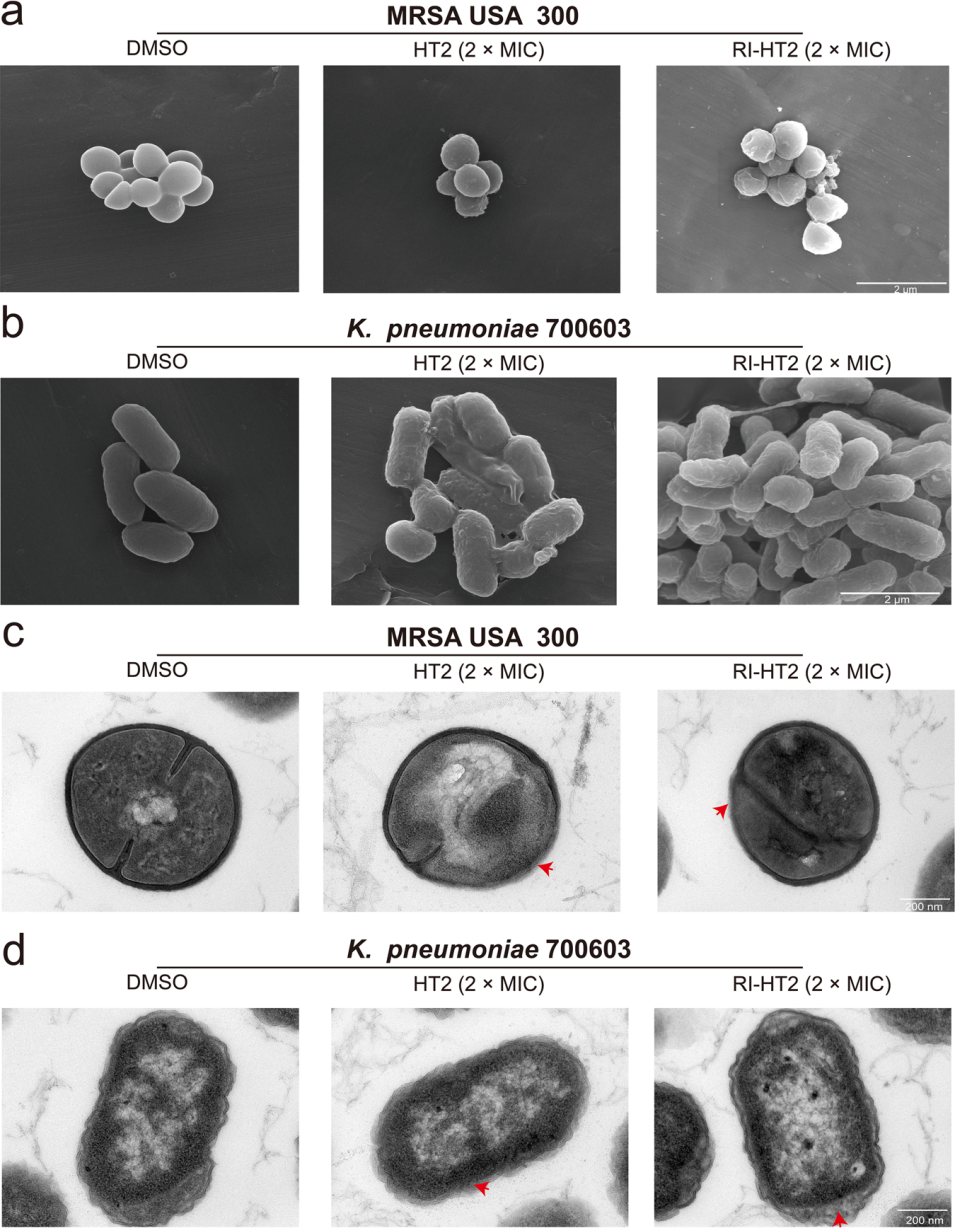
Membrane permeabilization confirmation by SEM and TEM studies. **a** SEM images of MRSA USA 300 treated with DMSO, HT2 and RI-HT2. **b** SEM images of *K. pneumoniae* ATCC 700603 treated with DMSO, HT2 and RI-HT2. **c** TEM images of MRSA USA 300 treated with DMSO, HT2 and RI-HT2. **d** TEM images of *K. pneumoniae* ATCC 700603 treated with DMSO, HT2 and RI-HT2. Scale bar, 2 µm (SEM), 200 nm (TEM).

The above results indicated that the peptides could cause damage to bacterial cell membranes. Scanning electron microscope (SEM) was used to further investigate the effect of peptides on bacterial cellular morphology. We observed that, in the absence of peptides, the normal bacteria MRSA USA 300 and *K. pneumoniae* ATCC 700603 appeared intact with a smooth surface. In contrast, the surface of MRSA USA 300 and *K. pneumoniae* ATCC 700603 became rough, shrunken, wrinkled and partially damaged upon treatment with HT2 and RI-HT2 (Fig. 5a, b).

To investigate the membrane integrity and intracellular material alterations of MRSA USA 300 and *K. pneumoniae* ATCC 700603 cells treated with HT2 and RI-HT2, samples were further observed by transmission electron microscopy (TEM) (Fig. 5c, d). Intact membrane envelopes and dense internal structures were observed in untreated bacterial cells. However, treatment with HT2 or RI-HT2 led to a significant change in the intracellular structure of MRSA USA 300 and *K. pneumoniae* ATCC 700603, and the cytoplasmic membranes became blurred and separated, and outflow of cytoplasm was observed. Thus, these results suggested that HT2 and RI-HT2 disrupt the integrity of bacterial cell membrane.

Taken together, our data consistently suggested that HT2 and RI-HT2 exhibit potent antibacterial activity by permeabilizing the bacterial membrane.

### Structural determination of HT2 and RI-HT2

Because of its parent peptide temporin-Shf exhibiting a well-defined amphipathic α-helical structure from residue Phe3 to Phe8 and a disordered dual-Phe motif in the presence of membrane mimics, we hypothesized that HT2 peptide could adopt a “tadpole-like” conformation consisting of α-helical structure from residues Phe2 to Phe7 and a hydrophobic tail from residue Phe8 to Phe9. It is known that short RI-peptides assume a 3D conformation very close to their corresponding L-forms^37^. To explore their secondary structure, we performed CD studies of HT2 and RI-HT2 (Fig. S3). The HT2 peptide was unstructured in aqueous solution but adopted helical structures in the presence of membrane mimetics. CD spectra of HT2 peptide in a membrane-mimetic environment such as 80 mM sodium dodecyl sulfonate (SDS) or 50% trifluoroethanol (TFE) presented a strong positive maximum band at 192 nm and an apparent negative band at 208 nm that are characteristics of α-helix secondary structure (Fig. S3a, b). This feature of CD spectra of HT2 was in line with the CD study of natural temporin-SHf^28^. In contrast, CD spectra of RI-HT2 peptide did not tend to form α-helix secondary structure in aqueous solution as well as in a membrane-mimetic environment (Fig. S3c–d).

**Fig. 6:**
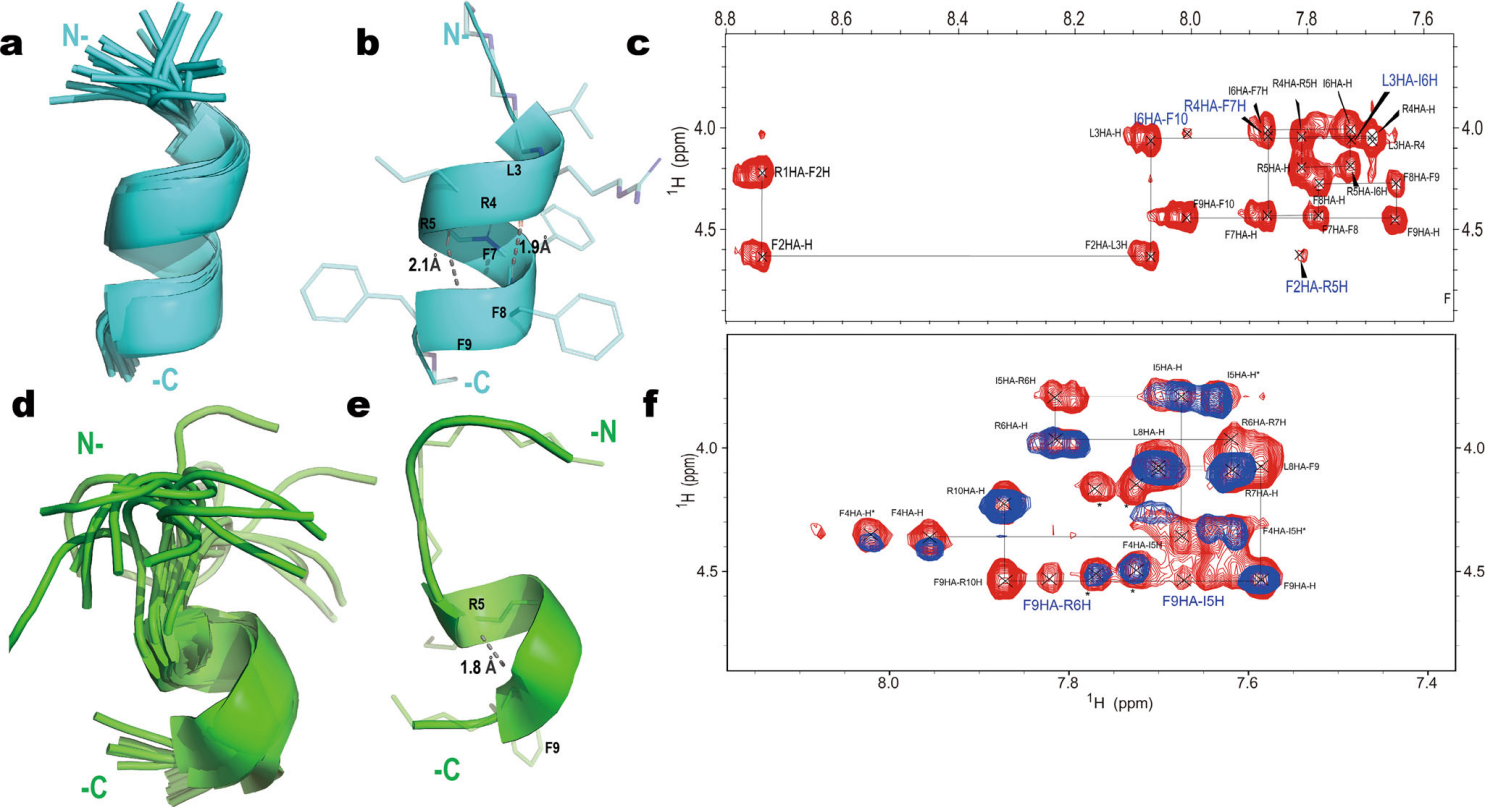
Solution NMR structures of HT2 (cyan) and RI-HT2 (green) in SDS micelles. **a**, **d** The backbone trace super position of the twenty lowest energy conformers of HT2 (cyan) and RI-HT2 (green). **b**, **e** Ribbon diagram of the lowest energy structure of HT2. The straight dashed lines in gray indicate hydrogen bonds, and the key residue are showed in sticks. **c**, **f** NOESY spectra showing sequential connection of HT2 and RI-HT2. The interaction network including F2HA-R5H, L3HA-I6H, R4HA-F7H, I6HA-F10H in HT2 (**c**) as well as F9HA-R6H and F9HA-I5H in RT-HT2 (**f**) were colored in blue. In (**f**), TOCSY spectra (blue) are overlaid in NOESY spectra (red). Locations mark with an asterisk (*) represent unassignment peaks.

Subsequently, the conformations of HT2 and RI-HT2 were investigated with NMR spectroscopy to provide an additional assessment of their conformational behavior in the presence of SDS micelles. Based on chemical shift assignments (Table 2 and Table S2), the TALOS program indicated that the Leu3 to Phe9 region of HT2 adopted an α-helical conformation, whereas Arg1, Phe2 in the N-terminus and Phe10 in the C-terminus appeared to be disordered without prediction. The interproton distance restraints were combined with dihedral angle restraints to determine the structure of the helical region of HT2 peptide. The root mean square deviation of all backbone atoms is 1.0 Å, and the structures do not display distance violations greater than 0.5 Å. In the calculated structures, segment 3–9 forms a well-defined helix, which is stabilized by an interaction network of Leu3, Arg4, Phe7 and Phe8 (PDB ID 8HVS) (Fig. 6a–c). Amide signals from D-Phe1, D-Phe2 and D-Arg10 were not observable in RI-HT2 NMR spectra. This is probably due to the high proton-solvent exchange rate of these residues, that are solvent-exposed, at the pH where the spectra have been recorded. The absence of long and medium range NOEs in the N-terminus of RI-HT2 results in a less well-defined structure in the N-terminus as compared to the HT2 peptide. In the final ensemble of RI-HT2 structures, the α-helix was found to be located at the C-terminal region between Ile5 and Leu8, suggesting that the amino acid configuration and the topological order introduce both dynamics and conformational changes in the structure (Fig. 6d–f).

**Table 2:** NMR and refinement statistics for HT2 peptide.

Protein		
NMR distance and dihedral constraints^a, b^		
Distance constraints^c^		
Total NOE	20	
Intra-residue	11	
Sequential (|*i* – *j* | = 1)	5	
Medium-range (|*i* – *j* | < 4)	4	
Long-range (|*i* – *j* | > 5)	0	
Total dihedral angle restraints		
ϕ	7	
ψ	7	
Number of structures used	20	
Structure statistics		
Violations (mean and s.d.)		
Distance constraints (Å)	0.531 ± 0.459	
Dihedral angle constraints (°)	0.559 ± 0.411	
Max. dihedral angle violation (°)	1.359	
Max. distance constraint violation (Å)	2.049	
Deviations from idealized geometry		
Bond lengths (Å)	0.001 ± 0.000	
Bond angles (°)	0.212 ± 0.004	
Impropers (°)	0.137 ± 0.006	
Average pairwise r.m.s. deviation (Å)		
Heavy	2.3	
Backbone	1.0	

^a^Analyzed for residues 1 to 10.

^b^There are 9 residues with conformationally restricting constraints.

^c^Calculated for all constraints for the given residues, using sum over r^-6.

Taken together, these results suggested that HT2 and RI-HT2 adopt a unique flexible “tadpole-like” conformation.

### Evaluation of peptide–membrane interactions by molecular dynamics simulations

**Fig. 7:**
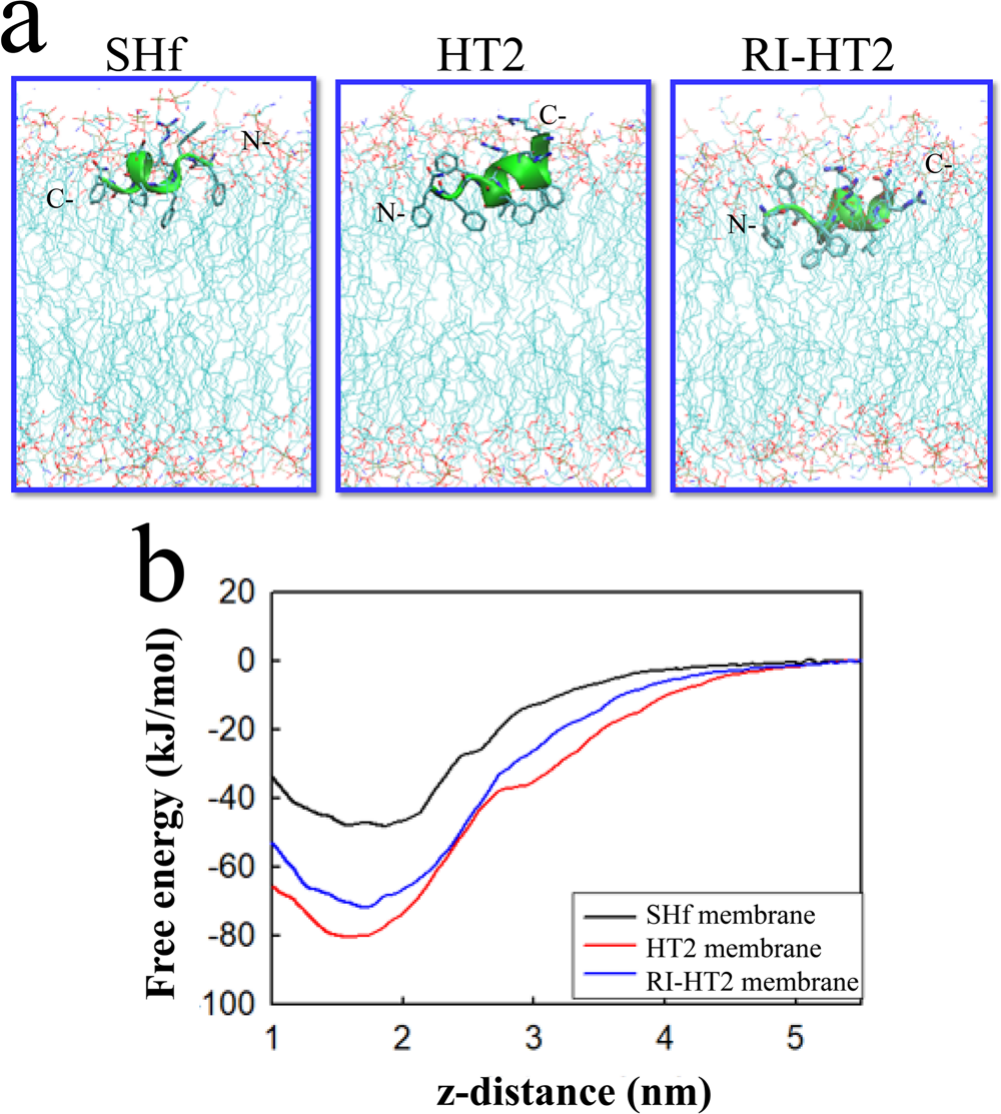
Interactions of model peptides with bacterial membrane. **a** Conformation of the peptides at the membrane-water interface. **b** Free-energy of peptide adsorption from the aqueous phase to the membrane phase constructed by umbrella sampling. The distance between the bilayer center and the center of mass of the compounds (z-distance) was used as the reaction coordinate.

To gain deeper insight into the mechanism of antibacterial activity of HT2 and RI-HT2, we used molecular dynamics (MD) simulations to study the interactions of temporin-SHf, HT2 and RI-HT2 with bacterial membranes. The models of bacterial membranes were constructed out of 1-palmitoyl-2-oleoyl-*sn*-glycero-3-phosphoethanolamine (POPE) and 1-palmitoyl-2-oleoyl-*sn*-glycero-3-phosphoglycerol (POPG) molecules at a ratio of 3:1. We first performed conventional MD to understand the mode of interactions of temporin-SHf, HT2 and RI-HT2 with the bacterial membranes (Table S3). We observed that all the peptides adopt helical conformations in the membrane environment, consistent with the conformational transition from disordered structure in aqueous phase to helical conformation in the membrane phase (Fig. S4). The longer sequences of HT2 and RI-HT2 when compared to temporin-SHf contribute to enhancing the helical contents of these analogs. We observed that all three peptides adopt a tadpole-like structure consisting of a helical segment and a disordered tail during the conventional MD (e.g., the last 600 ns). For the parent peptide temporin-SHf, the helical segment is from F3 to F8 and the N-terminal residues F1 and F2 adopt flexible conformations, consistent with a previous study^28^. In contrast, HT2 consists of a helical segment from the N-terminus from F2 to F7 or F8 and a flexible tail at the C-terminus. RI-HT2, with the reversed sequence of HT2, shifts its helical segment to the C-terminal region from F3 to F9 and a flexible tail at the N-terminal region (Fig. 7a). The difference in the helical region between HT2 and RI-HT2 arises from the fact that HT2 and RI-HT2 are retro-inverso peptides. We noticed that the helical region of HT2 shifted to N-terminus compared to the NMR structure whose helical region is located close to C-terminus. This could arise from the different micro-environments between simulations and experiments. Unlike macromolecules such as proteins, the conformations of short peptides are relatively dynamic and strongly depend on the micro-environments^38^. In MD simulations, we used a flat membrane consisting of a mixture of zwitterionic POPE and anionic POPG lipids, while in NMR experiments, the peptides fold on a negatively charged SDS micelle with curved surface. This could result in different interactions with the peptide, hence forming different conformations. Nevertheless, for all three peptides, the helical segment displayed a facial amphiphilic conformation with cationic residues facing aqueous phase and the hydrophobic residues facing lipid tails and the tails built from poly-aromatic residues. The helix of HT2 and RI-HT2 results in more favorable interactions with the membrane. As the helical segment is amphiphilic, the charged residues can engage strong electrostatic interactions with the head groups while the non-polar residues can perturb the lipid tail region of the membrane. In addition, the aromatic tails can engage hydrophobic interactions with the lipid tails, further perturbing the membrane structure.

A useful way to characterize the membrane activity of antimicrobial peptides is the interfacial activity model^39^, which attributes the antimicrobial activity of peptides to the ability of perturbing the membrane-water interface. Since the first step of membrane perturbation is the adsorption of the peptide onto the membrane, peptides that can reach a high concentration on membrane surface will have a high tendency to perturb the bacterial membrane. To understand the thermodynamics of peptide partitioning onto the membrane, we calculated the free energy of adsorption of the peptide from aqueous phase to the membrane phase as a function of peptide-membrane distance using umbrella sampling simulations (Fig. 7b). All the peptides displayed a free energy minimum around 1.7 nm, corresponding to the peptide being at the membrane-water interface. HT2 and RI-HT2 demonstrate more favorable free energies at the membrane-water interface than temporin-SHf, suggesting stronger interactions with the membrane. As HT2 and RI-HT2 carry two additional positive charges compared to temporin-SHf, clearly the favorable free energy of adsorption of HT2 arises from electrostatic interactions. As the electrostatic interactions are long-ranged compared to hydrophobic interactions, HT2 and RI-HT2 can accumulate onto the surface of the bacterial membrane up to a higher concentration than temporin-SHf. As a result, HT2 and RI-HT2 can induce more perturbations to the bacterial membrane, which is consistent with the interfacial activity model^39^.

### Peptides show low propensity to induce resistance

**Fig. 8:**
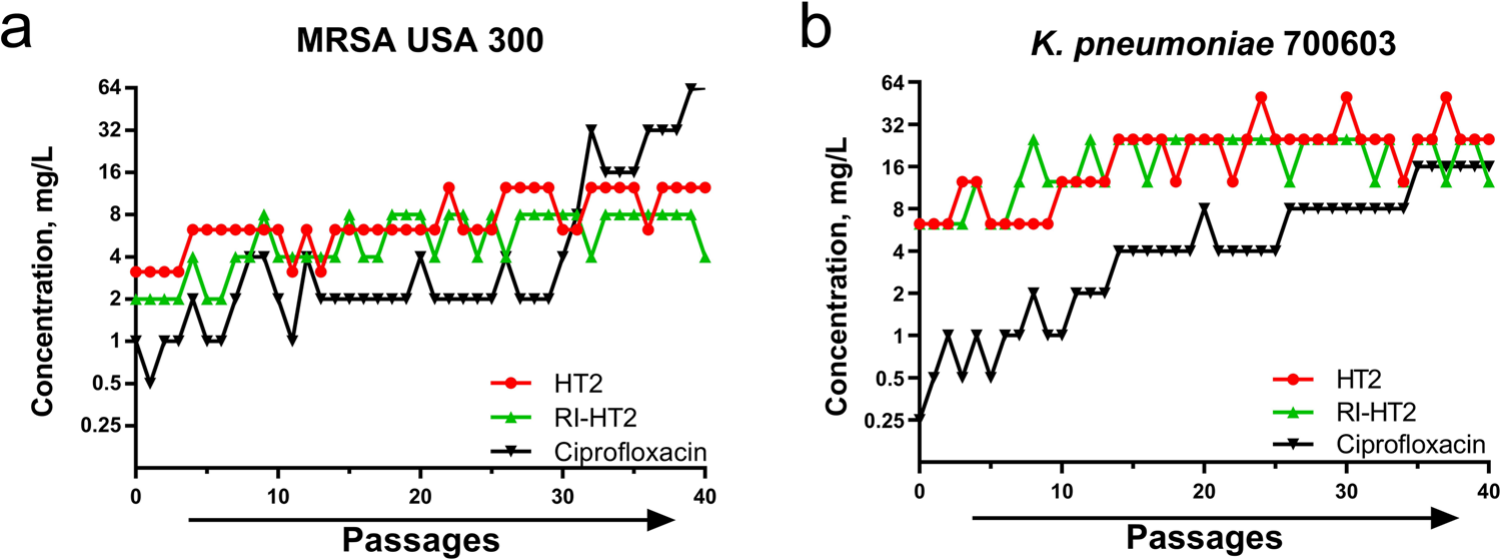
Serial passage resistance studies. Serial passage resistance induction of Ciprofloxacin, HT2 and RI-HT2 against MRSA USA 300 (**a**) and *K. pneumoniae* ATCC 700603 (**b**) was evaluated. We observed the development of drug resistance of MRSA USA 300 and *K. pneumoniae* ATCC 700603 in the presence of Ciprofloxacin (Cip), but not HT2, and RI-HT2 at sub-MIC levels.

Development of resistance is a great challenge that hampers antibiotic development and eradication of nosocomial pathogens. We therefore evaluated the resistance development of HT2 and RI-HT2 through serial passaging in the presence of sub-inhibitory concentrations of the peptides. With exposure to the classic antibiotic ciprofloxacin, the MICs of MRSA USA 300 and *K. pneumoniae* ATCC 700603 had no significant changes in the first 20 and 30 generations, but showed obvious resistance with a 64-fold increase in MIC occurred after 40 passages (Fig. 8). However, no significant changes were observed for both HT2 and RI-HT2, with only a four-fold change after 40 passages. Therefore, HT2 and RI-HT2 show low propensity to induce resistance.

## Discussion

The distinct antimicrobial activity of antimicrobial peptides is reflected in their unique folded structures and interactions with bacterial membranes. We propose a unique “tadpole-like” antimicrobial peptide consisting of an amphipathic α-helical head and a flexible aromatic tail. Starting from the natural antimicrobial peptide temporin-SHf, a series of ultrashort “tadpole-like” antimicrobial peptides were synthesized via a rational design approach taking into account the net charge, amphipathicity, hydrophobicity and topological rearrangement.

We have established a detailed structure-activity and structure-toxicity relationship of these “tadpole-like” antimicrobial peptides. First, the flexible tail is essential for antimicrobial activity. The addition of one or two Phe residues to the N-terminus hydrophobic tail of temporin-SHf results in increased antimicrobial activity. Second, electrostatic interactions significantly affect the antimicrobial activity as increasing positive charges enhance antimicrobial activity. For example, substitution of Ser5 with Arg is beneficial for antibacterial activity, whereas the substitution with Glu has a detrimental effect on the antimicrobial activity. Third, topological rearrangement has an effect on the antimicrobial activity. Reversing topological order of peptide FF-SHf to HT2 resulted in further separation of hydrophobic residues and charged residues. As a result, HT2, with a higher hydrophobic moment, displayed increased antimicrobial activity. Fourth, the attachment of additional Arg residue to N-terminus enhances the hydrophobic moment, thus resulting in increased antimicrobial activity. Fifth, balance between the number of positive charges and the number of hydrophobic residues is important for antimicrobial activity. For the peptides in this study, a net charge of +4 results in the best antimicrobial activity. However, a significant increase of hemolytic activity and cytotoxicity was observed beyond +4 net charge. Sixth, HT2 and its retro-inverso form RI-HT2 show similar antimicrobial activity, but the former has better stability. Overall, we demonstrate that, by tuning these physicochemical parameters and topological rearrangements, it is possible to design safe synthetic peptides with enhanced antimicrobial potency.

Our systematic studies led to a series of novel ultrashort peptides with potent antimicrobial activity. These peptides, including F-SHf(R^5^), F-SHf(R^5^)-R, F-SHf(R^5^)-RR, HT1–5 and RI-HT2, displayed potent activity against a range of Gram-positive and Gram-negative bacteria. In particular, HT2–5 and RI-HT2 were effective against the high priority pathogens as defined by WHO, such as multi-drug resistant *S. aureus*, *E. coli*, *A. baumannii* and *K. pneumoniae*, which were not affected by the native parent temporin-SHf. Neither HT2 nor RI-HT2 showed toxicity against mammalian cells at 100 μM, suggesting a wide activity-toxicity therapeutic window. Both peptides permeabilize bacterial membranes of MRSA USA 300 and *K. pneumoniae* ATCC 700603 in the scanning and transmission electronic microscopic experiments, validating the membrane targeting mechanism.

HT2 exhibits helical structures in the presence of membrane mimetics, but it is unstructured in a water environment. The NMR data clearly indicate that HT2 adopts a well-defined helix encompassing residues 3–9 whereas RI-HT2 also adopts a helix starting at residue Ile5 and ending at residue Leu8 in a membrane-mimetic SDS environment. Both peptides display “tadpole-like” conformations consisting of an α-helical head and a disordered tail. Indeed, MD simulations of the peptides in atomistic bilayer models showed that HT2 and RI-HT2 predominately adopt a helical conformation on bacterial membranes, with the N-terminal being helical/C-terminal being disordered and N-terminal being disordered/C-terminal being helical, respectively. Upon being adsorbed on the membrane surface, the parent peptide temporin-SHf lies almost parallel to the membrane surface because the N-terminus and C-terminus have a positive charge and engage in electrostatic interactions with the head groups. In contrast, the positive charges of HT2 and RI-HT2 are mainly located at the helical segments, while the tail segments are hydrophobic. The high charge density of the helical segments can engage stronger electrostatic interactions with the head groups, while the hydrophobic residues can penetrate into the lipid tail regions of the membranes. Moreover, the higher positive charges enable HT2 and RI-HT2 to electrostatically interact more favorably with the membrane head groups than temporin-SHf, as shown by the more favorable free energies of adsorption of the peptides on to the membrane surfaces. This suggests that compared to temporin-SHf, HT2 and RI-HT2 can accumulate on the bacterial membrane surfaces up to higher concentrations and can eventually induce larger membrane perturbations, which is consistent with the interfacial activity model^39^.

Natural AMPs have diverse secondary structures including five main categories, α-helix, β-sheet, β-hairpin or loop, αβ mixed and extended^30,40^. Among these structures, amphipathic α-helix is one of the most common types of AMPs in nature and has been reported to exert their effects via membrane-pore-forming mechanisms^10,41,42^. We developed a class of AMPs displaying a “tadpole-like” conformation consisting of an amphipathic α-helical head and flexible hydrophobic tail. These unique “tadpole-like” conformations can simultaneously perturb both the head groups and the lipid tails of the membranes, thereby showing increased antimicrobial activities compared to a pure α-helical peptide alone. In addition, a topological rearrangement of “tadpole-like” peptide using circular permutation greatly enhanced antimicrobial activity. Therefore, peptides with “tadpole-like” conformations could be a useful scaffold for the design of new antimicrobial peptides.

In conclusion, our study not only leads to a series of ultrashort peptide analogs with potent and broad-spectrum antimicrobial activity, but also provides a new strategy for the rational design of “tadpole-like” antimicrobial peptides to tackle the threat of antimicrobial resistance.

## Methods

### Peptide synthesis and purification

All peptides were synthesized employing an Fmoc-based solid phase peptide synthesis strategy using Rink-Amide resin^43^. Briefly, Rink-Amide resin (initial loading 0.57 mmol/g) was swelled in anhydrous DCM and washed by DMF. The subsequent N-terminal peptide chain elongation was achieved by standard Fmoc strategy employing the commercially available amino acid building blocks. Fmoc group cleavage was achieved by shaking the resin twice for 10 min with a solution of piperidine/DMF (1:4 v/v). Double coupling was performed for 2 h (4.0 eq. amino acid, 4.0 eq. HCTU and 8.0 eq. DIPEA in DMF). After elongation of the peptide chain, the Fmoc group was removed and the resin was washed with DCM and dried overnight in vacuo. Cleavage from the solid support was performed by treating the resin with the cocktail (95 % TFA in water) for 3 hours. After cleavage, the solvent was evaporated under reduced pressure and the resulting crude was slowly precipitated using an ice-cold diethyl ether solution. The precipitate was centrifuged, dissolved in water and subjected to lyophilization. Peptides were obtained after purification by preparative HPLC using a C18 column, and characterized by high resolution-mass spectroscopy (HR-MS).

Chemicals were obtained from Merck, GL Biochem or Aladdin and used without further purification. Analytical HPLC-MS data were recorded on a Hitachi HPLC (Primaide) with a C18 column (Bondysil C18, 5 µm, 250 mm × 5.6 mm). High resolution mass spectra (HR-MS) of peptides were measured on a Thermo Orbitrap coupled to a Thermo Accela HPLC system using electrospray ionization (ESI). The preparative HPLC purifications were carried on a Hitachi HPLC (Primaide) using a reversed-phase C18 (Bondysil C18), 5 µm, 250 mm × 10 mm, flow 3.0 mL/min, from 10% A to 100% A over 20 min (A = acetonitrile + 0.1% TFA; B = water + 0.1% TFA).

### Bacterial strains and growth media

The bacterial strains used in this study included the Gram-positive bacteria methicillin resistant *Staphylococcus aureus* (MRSA) (MRSA ATCC 43300 and 2 clinically isolated strains presented as MRSA USA 300 and MRSA 3393), Gram-negative bacteria *Escherichia coli* (*E. coli* ATCC *25922*, and 1 clinically isolated strain presented as *E. coli* S), *Klebsiella pneumoniae* (*K. pneumoniae* ATCC 700603, and 1 clinically isolated strain presented as *K. pneumoniae* 3026), and *Acinetobacter baumannii* (2 clinically isolated strains presented as *A. baumannii* 1318 and *A. baumannii* 1337). Bacteria were cultivated in Luria-Bertani (LB) broth.

### Antimicrobial activity

The Clinical and Laboratory Standards Institute (CLSI)-defined broth microdilution testing method (M07, 11th edition, 2018) was used to determine MICs for peptides. Briefly, the concentrations of the peptides and ciprofloxacin in the assay wells ranged from 100 μM to 0.01 μM as a 11-point dose response plated as a 2-fold dilution series across the wells of the plate in duplicate for each independent biological replicate. Bacterial susceptibility assays were performed in Cation-Adjusted Mueller Hinton Broth (CAMHB), and using a final cell density of 5 × 10^6^ CFU/mL for bacteria^36^. Final assay volumes were 100 μL per well with MICs determined by measuring absorbance at 595 nm with a microplate reader following 18–20 h incubation at 37 °C. All experiments were carried out with a minimum of six replicates (*n* ≥ 6).

### MTT assay

To determine the cytotoxicity of the peptides, we used the MTT (3-(4,5)-dimethylthiahiazo (-z-y1)-3,5-di-phenytetrazoliumromide) dye reduction assay against HEK293T cells. In brief, the 100 μL of cells in 96-well plate were incubated for 24 h at 37 °C, 5% CO_2_, and then peptide solutions (concentrations ranging from 100 to 0.39 μM) were added and further incubated for 72 h. MTT solution (5 mg/mL) was added and further incubated for 4 h. After incubation, the precipitated MTT formazan crystal was dissolved by addition of dimethyl sulfoxide (DMSO). Absorbance at 595 nm was measured using a microplate reader, using Prism 8 following curve fitting.

### Hemolysis assay

To evaluate the hemolysis effects of peptides, red blood hemolysis was performed as described with minimum modifications^44^. Sterile defibrated sheep blood was purchased from Guangzhou Hongquan Biotechnology Co., LTD. Blood cells were adjusted to 2 × 10^9^ cells/mL in phosphate-buffered saline (PBS, pH 7.4). In total, 98 μL sheep red blood cell suspension was incubated with 2 μL of peptides at different concentrations ranging from 3.13 to 100 µM. After incubation for 1 h at 37 °C, the cells were centrifuged and the absorbance of the supernatant was measured at 450 nm. The value for “zero hemolysis” was determined using sterile PBS, while 100% hemolysis was established using 1.5% (v/v) Triton X-100. Hemolysis of testing sample was calculated as the percentage of Triton X-100-induced hemolysis.

### Time-kill assay

Time-kill assays were done in tubes (in 1.5 mL of LB) and antibiotics at the 1 ×, 2 ×, 4 × and 8 × MIC. Tubes were inoculated (5 × 10^6^ CFU/mL) with MRSA USA 300 and *K. pneumoniae* ATCC 700603 and incubated at 37 °C under shaking. Serial dilutions of the cultures were plated at each desired time point (0, 1, 3, 6, and 24 h), using a multichannel pipette as follows: in line 1 of a 96-well plate, 100 µL of bacterial suspension was added, while 90 µL of 0.9% sterile saline per well was added to the other lines. At selected time points, 100 µL of bacterial suspension was transferred from the time-kill assay plate to the first line. The wells were further diluted 1 in 10 (0.9% saline) for the appropriate number of dilutions and 10 μL of each dilution was spotted in duplicate onto MH agar plates, then incubated overnight at 37°C. The colonies in each spot were counted and used to calculate the number of viable CFU/mL remaining in the original culture by considering the dilution factors.

Time-kill assay of *S. aureus* ATCC 25923 was done in tube (in 1.5 mL of LB) and antibiotics at 16 × MIC. Tube was inoculated (5 × 10^6^ CFU/mL) with *S. aureus* ATCC 25923 and incubated at 37°C under shaking. Bacterial concentration was measured at each time point (0, 1, 2, 2.5, 3, 3.5, 4, 5, 6 and 7 h) by measuring absorbance at 600 nm with a microplate reader.

### Stability analysis

The stability of peptides in different media was assessed using the MIC assay described above. Here, the stability of HT2 and RI-HT2 in serum, salt, and different pH were analyzed using methods previously described with some modifications^45^. For serum stability, a solution of 10% or 20% fetal bovine serum (FBS) was made by diluting fetal bovine serum with phosphate buffer saline (PBS). HT2 and RI-HT2 were added for a final concentration of 160 µg/mL of peptide in the FBS solution, and incubation 2 h. Suspension containing no peptide was considered the negative control. The MIC of HT2 and RI-HT2 against MRSA USA 300 and *K. pneumoniae* ATCC 700603 were measured. For the acid stability assays, the peptides were incubated with different pH values of PBS (pH = 5.5 or 6.4) at 37 °C. These peptide mixtures were then serially diluted in a 96-well plate for the evaluation of antimicrobial activity against bacterial strains. Similarly, for salt stability, the peptides were assessed for their antimicrobial activity against MRSA USA 300 and *K. pneumoniae* ATCC 700603 in the presence of 150 mM NaCl.

### Membrane depolarization assay

MRSA USA 300 and *K. pneumoniae* ATCC 700603 *w*ere inoculated into 5 mL of LB and shaken at 37°C and 225 rpm to OD600 ≈ 0.5 (i.e., mid-logarithmic phase). Bacteria were collected by centrifugation at 8000 rpm for 5 min, and washed twice with assay buffer (5 mM HEPES, 20 mM glucose, 10 mM KCl, pH 7.2). Bacteria were resuspended in the assay buffer to OD_595_ ≈ 0.1. DiSC_3_(5) was added to the cells at final concentration of 0.3 µM, wrapped with aluminum foil, and incubated at 37°C, until a stable fluorescence was observed. Two microliter of compound or DMSO were transferred into 96-well black walled polystyrene plates, to which was added 98 µL per well of the dye-saturated bacterial cells to initiate the reaction. The final concentration of HT2 and RI-HT2 were 1 ×, 2 ×, 4 × and 8 × MIC. Plates were immediately read on an Envison at λex = 622 nm/ λem = 670 nm with a kinetic program (reading every 1 min for 10 min) until the maximal intensity achieved was observed.

### Membrane permeability

Fluorescent probe SYTOX^TM^ Green (Invitrogen/ Life Technologies, S7020) was used to analyze the effect of HT2 and RI-HT2 on membrane permeability of MRSA USA 300 and *K. pneumoniae* ATCC 700603 as described elsewhere^46^. SYTOX^TM^ Green is a DNA binding dye that is fluorescent when bound to nucleic acids and only enters cells with compromised plasma membranes. MRSA USA 300 and *K. pneumoniae* ATCC 700603 were cultured in LB broth to mid log phase at 37 °C and washed with PBS and resuspended (OD595 ≈ 0.1) in the same buffer. SYTOX^TM^ Green was added into the cell suspension with a final concentration of 5 μM and being kept in dark for 20 min. 98 µL pre-treated cell suspension was transferred into wells of 96-well glass black plates. Then, 2 µL of compound or DMSO were added to initiate the reaction. The final concentrations of HT2 and RI-HT2 were 1 ×, 2 ×, 4 × and 8 × MIC. Fluorescence strength was measured with excitation/emission wavelength of 485/528 nm every 5 min for 60 min.

### Drug resistance assay

MRSA USA 300 and *K. pneumoniae* ATCC 700603 were employed to assess the development of resistance. From inoculated microtiter panels, an aliquot of the well with the highest concentration permitting growth was taken and back diluted in fresh media to a turbidity of a 0.5 McFarland standard. This suspension was then further diluted and used to inoculate a fresh MIC panel resulting in a final concentration of 5 × 10^6^ CFU/mL. Panels were incubated according to CLSI guidelines (M07, 11th edition, 2018), MICs were recorded and the next inoculum was prepared from the well containing the highest concentration of drug that allowed growth in identical fashion as described above.

### Scanning electron microscopy

Mid-log phase bacteria (MRSA USA 300 and *K. pneumoniae* ATCC 700603) grown in LB broth were adjusted to a density of 5 × 10^6^ CFU/mL. HT2 and RI-HT2 were added to bacterial suspensions at a final concentration of 2 × MIC and incubated for 60 min at 37°C under constant shaking. All samples were centrifuged at 8000 rpm for 5 min, washed and then resuspended in PBS. Bacterial cells were fixed with 2.5% glutaraldehyde, and then incubated at 37°C on a glass slide for 20 min. Samples were dehydrated by passing through gradient ethanol (10%, 30%, 50%, 70%, 90%, and 100%) for 5 min at each concentration. Slides were dried with an automatic critical point drying instrument (Leica EM CPD300), and a small amount of gold was sputtered onto the samples using a sputter coater system to avoid charging in the microscope. Finally, the bacteria were visualized under a field-emission scanning electron microscopy (FESEM) device (Sirion 200).

### Transmission electron microscopy

The processing method of the samples for transmission electron microscopy (TEM) was the same as the treatment for SEM. The samples were then prefixed with 2.5% glutaraldehyde at 4°C overnight, post-fixed with osmium tetroxide for 70 min, and washed twice with PBS (pH 7.4). Subsequently, the samples were dehydrated for 10 min in a graded ethanol series (30, 50, 70, 90, and 100%) followed by 10 min in a mixture of absolute ethanol and acetone (1:1, v/v) and 10 min in absolute acetone. The specimens were transferred to a mixture of absolute acetone and epoxy resin (1:1, v/v) for 30 min and then to pure epoxy resin overnight at a constant temperature. Finally, the samples were sectioned using an ultramicrotome, stained with uranyl acetate and lead citrate, and then observed by TEM (Hitachi H-7650, Japan).

### Circular dichroism

Circular dichroism (CD) spectroscopy was acquired using an Applied Photophysics spectrometer (CHIRASCAN) as described in our previous study^47^. Peptides were dissolved into trifluoroethanol (TFE) to prepare a stocking solution of 7.5 mM. For CD analysis, peptides were diluted to 100 μM in different concentrations of TFE or sodium dodecyl sulfonate (SDS). Peptide samples were filled in 0.1 cm quartz cuvette and scanned from 195 nm to 260 nm at 20°C, with the scanning speed at 20 nm/min and CD spectra were accumulated 3 times. CD spectra of peptide samples were subtracted from the spectra of buffer and smoothened using an FFT filter. The unit of millidegree (***θ***) was converted to mean residue molar ellipticity ***[θ]***_***MRw***_,_***λ***_ with the following Eqs. (1) and (2).

1 $${\left[\theta \right]}_{{MRW},\lambda }=\frac{{\theta }_{\lambda }}{10\times d\times {c}_{r}}$$

2 $${c}_{r}=\frac{c}{{Mw}/n}$$

where ***λ*** is the wavelength, ***θ*** is the observed ellipticity in degrees, ***c***_***r***_ is the mean residue molar concentration in g/mol, ***d*** is the path length in cm, ***Mw*** is the molecular weight of the peptide, ***c*** is the concentration in g/mL and ***n*** is the number of amino acid residues.

### Nuclear magnetic resonance (NMR) spectroscopy and structure calculation

All NMR experiments for structure calculations were carried out at 313 K using 700 MHz Bruker Avance NMR spectrometers equipped with a 5-mm cryogenic probe. The HT2 and RI-HT2 peptides were dissolved to 1.4 mM in 10% D_2_O/ 90% H_2_O (v/v) containing 80 mM SDS-d25. Two-dimensional (2D) homonuclear ^1^H experiments, including total correlation spectroscopy (TOCSY) and nuclear Overhauser enhancement spectroscopy (NOESY), and 2D ^13^C − ^1^H experiment heteronuclear single quantum coherence spectroscopy (HSQC) were performed using conventional pulse sequences. Meanwhile, NOESY experiments with a mixing time (300 ms) reduced the spin diffusion effect. All NMR spectra were processed using NMRPipe^48^ and analyzed using NMRFAM-Sparky^49^. The restraints for backbone torsion angles were derived from chemical shifts using the TALOS software program^50^. The interproton distance restraints were estimated from NOE cross-peak volumes by manual and iterative refinements. The solution structure was calculated with CYANA 2.1 using standard parameters^51^, the final 20 conformers from 100 calculated ensembles with the lowest energy were deposited in the Protein Data Bank (PDB, ID: 8HVS). Structural statistics and global structure quality factors were computed using PSVS version 1.5 and PROCHECK-NMR^52,53^ (Table 2).

### Molecular dynamics simulations

Atomistic molecular dynamics (MD) simulations were carried out to study the mode of interactions of the three peptides temporin-SHf, HT2 and RI-HT2 with a model bacterial membrane constructed out of 96 zwitterionic lipids POPE and 32 anionic lipids POPG. This model can capture the main structural features of bacterial membrane and has been widely used in previous studies^54–57^. The coordinates of the membrane were taken from our previous studies^58^. The peptide in helical conformation was first placed close to the membrane and then solvated with water molecules. Counter ions were added to neutralize the system. To remove bad atomic contacts, 500 steps of energy minimization using steep descent algorithm was performed, followed by 100 ps NPT simulations to relax the system. Next a three-step production MD simulation was carried out for each peptide. Due to the complex free energy landscape of the peptide-membrane system, the time scale to efficiently sample the conformational space is considerably long. To accelerate the conformational sampling, in the first step, we applied multiple cycles of simulated annealing for 200 ns with elastic network (ELN) applied to the alpha-carbon of each residue to maintain a helical conformation. In the second step, the ELN was removed to relax the peptide conformation in the membrane environment. In the third step, a normal MD simulation without ELN and SA was carried out for 600 ns, resulting in 1000 ns simulations for each peptide. Each simulated annealing cycle was run for 10 ns, with the temperature of the system linearly increased from 310 to 350 K in the first 3 ns, maintained at 350 K for 4 ns and decreased to 310 K in another 3 ns. Increasing temperature in the membrane system has been used to study the mode of action of helical peptides by us and others^59,60^.

To understand the membrane affinity of the three model peptides, the free energy of adsorption of the two peptides on bacterial and mammalian membranes were carried out using umbrella sampling^61^. The distance between the center of mass of the peptide and the bilayer center was chosen as the reaction coordinate. In each umbrella sampling, a total of 28 window simulations spanning the distance from 1.05 to 5.1 nm were carried out. Each window simulation was run for 200 ns, with the last 105 ns used for constructing the free energy profile using weighted histogram analysis method (WHAM)^62^.

As the NMR revealed helical conformations for each peptide, the initial structures of the two peptides were modeled in helical conformations, which were generated using Discovery studio. CHARMM36m force field was used to model both the peptide and the membranes, and water molecules were modeling using CHARMM modified TIP3P model^63^. The topology of the peptide and lipids were generated using CHARMM-GUI^64^. In each simulation, the LJ interactions were calculated using a cutoff of 1.2 nm, with a force-switch between 1.0 nm and 1.2 nm. The short-range electrostatic interactions were calculated with a cutoff of 1.2 nm while the long-range electrostatic interactions were computed using PME^65^. During the MD simulations, the covalent bonds involving hydrogen atoms were constrained using the LINCS algorithm, enabling a time step of 2 fs to be used. The simulations were run in NPT ensemble, with temperature maintained at 310 K using Nose-Hoover method and pressure maintained using Parrinello-Rahman method with semi-isotropic pressure coupling. All simulations were carried out using GROMACS 2021^66^.

### Reporting summary

Further information on research design is available in the Nature Portfolio Reporting Summary linked to this article.

### Supplementary information

Supplementary Information

Description of Additional Supplementary Files

Supplementary Data 1

Reporting Summary

## Data Availability

The NMR structure of HT2 is available in the Protein Data Bank (PDB) under the accession code 8HVS. The original numerical data for the graphs are provided in Supplementary Data 1. All other data are available from the corresponding authors upon reasonable request.
