# Supplementary material for: Design and evaluation of tadpole-like conformational antimicrobial peptides
Source: Commun Biol. 2023 Nov 18;6:1177. doi: 10.1038/s42003-023-05560-0 (PMC10657444; doi:10.1038/s42003-023-05560-0)
Supplement: Supplementary file 1 — Supplementary Information [file 42003_2023_5560_MOESM1_ESM.pdf]

Supplementary Information for

# Design and evaluation of tadpole-like conformational antimicrobial peptides

Ziyi Tang[1,#], Wuqiao Jiang[2,#], Shuangli Li[4,#], Xue Huang[2], Yi Yang[2], Xiaorong Chen[2], Jingyi Qiu[1], Chuyu Xiao[2], Ying Xie[2], Xu Zhang[4], Jianguo Li[5,6], Chandra Shekhar Verma[5,7,8], Yun He[3,*], Aimin Yang[2,*]

[#]These authors contributed equally to this work

*Correspondence:

yun.he@cqu.edu.cn (Y. He)

aimin.yang@cqu.edu.cn (A. Yang)

**This PDF file includes:**

**Table S1 to S3**

**Figures S1 to S4**

**Table S1. Mass characterization and purity determination of peptides.** In peptide sequences, uppercase letters indicate L-amino acids, whereas the lowercase letters indicate D-amino acids.

| No. | peptide | sequence | MS form | $M_w$ calculated | $M_w$ found | purity (%) |
|---|---|---|---|---|---|---|
| 1 | Temporin-SHf | FFFLSRIF-$NH_2$ | $C_{57}H_{79}N_{12}O_9$ $[M+H]^+$ | 1075.61 | 1075.60 | 96.5 |
| 2 | SHfΔ1F | FFLSRIF-$NH_2$ | $C_{48}H_{70}N_{11}O_8[M+H]^+$ | 928.54 | 928.40 | 96.4 |
| 3 | SHfΔ2F | FLSRIF-$NH_2$ | $C_{39}H_{61}N_{10}O_7[M+H]^+$ | 781.47 | 781.50 | 99.4 |
| 4 | OA-SHf | OA-FLSRIF-$NH_2$ | $C_{47}H_{75}N_{10}O_8[M+H]^+$ | 908.18 | 908.20 | 98.7 |
| 5 | SHf($Y^1$) | YFFLSRIF-$NH_2$ | $C_{57}H_{79}N_{12}O_{10}[M+H]^+$ | 1091.60 | 1091.55 | 95.4 |
| 6 | SHf($Y^2$) | FYFLSRIF-$NH_2$ | $C_{57}H_{79}N_{12}O_{10}[M+H]^+$ | 1091.60 | 1091.50 | 96.5 |
| 7 | Y-SHf | YFFFLSRIF-$NH_2$ | $C_{66}H_{88}N_{13}O_{11}[M+H]^+$ | 1238.67 | 1238.65 | 95.7 |
| 8 | Y-SHf($Y^1$) | YYFFLSRIF-$NH_2$ | $C_{66}H_{88}N_{13}O_{12}[M+H]^+$ | 1254.67 | 1254.60 | 97.9 |
| 9 | Y-SHf($Y^2$) | YFYFLSRIF-$NH_2$ | $C_{66}H_{88}N_{13}O_{12}[M+H]^+$ | 1254.67 | 1254.65 | 98.1 |
| 10 | YY-SHf | YYFFFLSRIF-$NH_2$ | $C_{75}H_{97}N_{14}O_{13}[M+H]^+$ | 1401.74 | 1401.70 | 95.7 |
| 11 | FF-SHf | FFFFFLSRIF-$NH_2$ | $C_{75}H_{97}N_{14}O_{11}[M+H]^+$ | 1369.75 | 1369.60 | 96.2 |
| 12 | SHf($E^5$) | FFFLERIF-$NH_2$ | $C_{59}H_{81}N_{12}O_{10}[M+H]^+$ | 1117.62 | 1117.60 | 98.6 |
| 13 | Y-SHf($R^5$) | YFFFLRRIF-$NH_2$ | $C_{69}H_{95}N_{16}O_{10}[M+H]^+$ | 1307.74 | 1307.75 | 97.4 |
| 14 | F-SHf($R^5$) | FFFFLRRIF-$NH_2$ | $C_{69}H_{95}N_{16}O_9[M+H]^+$ | 1291.75 | 1291.80 | 98.5 |
| 15 | FF-SHf($R^5$) | FFFFFLRRIF-$NH_2$ | $C_{78}H_{104}N_{17}O_{10}[M+H]^+$ | 1438.82 | 1438.80 | 98.0 |
| 16 | F-SHf($R^5$)-R | FFFFLRRIFR-$NH_2$ | $C_{75}H_{107}N_{20}O_{10}[M+H]^+$ | 1447.85 | 1447.90 | 98.4 |
| 17 | F-SHf($R^5$)-RR | FFFFLRRIFRR-$NH_2$ | $C_{81}H_{119}N_{24}O_{11}[M+H]^+$ | 1603.95 | 1603.90 | 97.9 |
| 18 | F-SHf($dR^5$) | FFFFLrRIF-$NH_2$ | $C_{69}H_{95}N_{16}O_9[M+H]^+$ | 1291.75 | 1291.80 | 97.5 |
| 19 | F-SHf($R^5$, $dR^6$) | FFFFLRrIF-$NH_2$ | $C_{69}H_{95}N_{16}O_9[M+H]^+$ | 1291.75 | 1291.80 | 98.5 |
| 20 | F-SHf($dR^{5,6}$) | FFFFLrrIF-$NH_2$ | $C_{69}H_{95}N_{16}O_9[M+H]^+$ | 1291.75 | 1291.85 | 98.4 |
| 21 | HT1 | FLRRIFFFF-$NH_2$ | $C_{69}H_{95}N_{16}O_9[M+H]^+$ | 1291.75 | 1291.80 | 98.1 |
| 22 | HT2 | RFLRRIFFFF-$NH_2$ | $C_{75}H_{107}N_{20}O_{10}[M+H]^+$ | 1447.85 | 1447.85 | 99.7 |
| 23 | HT3 | RRFLRRIFFFF-$NH_2$ | $C_{81}H_{119}N_{24}O_{11}[M+H]^+$ | 1603.95 | 1604.00 | 97.7 |
| 24 | HT4 | RFLRRIFRFFF-$NH_2$ | $C_{81}H_{120}N_{24}O_{11}[M+2H]^{2+}$ | 802.48 | 802.50 | 96.8 |
| 25 | HT5 | RFLRRIFKFFF-$NH_2$ | $C_{81}H_{119}N_{22}O_{11}[M+H]^+$ | 1575.94 | 1576.60 | 97.1 |
| 26 | D-HT2 | rflrriffff-$NH_2$ | $C_{75}H_{107}N_{20}O_{10}[M+H]^+$ | 1447.85 | 1447.90 | 98.1 |
| 27 | RI-HT2 | ffffirrlfr-$NH_2$ | $C_{75}H_{107}N_{20}O_{10}[M+H]^+$ | 1447.85 | 1447.90 | 97.4 |

**Table S2. The assigned chemical shift of HT2 peptide**

| | H | HA | HB | HG | HD1 | HE | HZ |
|---|---|---|---|---|---|---|---|
| **R1** | NA | 4.22 | 2.23/2.08 | 1.85/1.85 | 3.34/3.34 | 7.26 | |
| **F2** | 8.74 | 4.63 | 3.33/3.24 | | 7.35/7.35 | NA/NA | NA |
| **L3** | 8.07 | 4.06 | 1.87/1.78 | 1.59 | 1.03/0.96 | | |
| **R4** | 7.68 | 4.04 | 1.95/1.95 | 1.72/1.72 | 3.28/3.28 | 7.18 | |
| **R5** | 7.81 | 4.19 | 1.95/1.95 | 1.75/1.75 | 3.25/3.25 | NA | |
| **I6** | 7.73 | 3.99 | | 1.56/1.56/1.21 | 0.93 | | |
| **F7** | 7.85 | 4.43 | 3.19/3.09 | | 7.14/7.14 | NA/NA | NA |
| **F8** | 7.78 | 4.27 | 3.05/2.93 | | 6.84/6.84 | 7.23 | 7.04 |
| **F9** | 7.65 | 4.45 | 3.33/2.92 | | 7.46/7.46 | NA/NA | NA |
| **F10** | 8.02 | 4.44 | 3.22/3.10 | | 7.31/7.31 | NA/NA | NA |

**Table S3. Details of each simulation system**

| Peptide | Lipid | Water | Ions | Three-stage procedure |
|---|---|---|---|---|
| **SHf** | 108 POPE + 36 POPG | 7927 | 34 NA + ions | 1: Simulated annealing and ELN (0-200 ns);<br>2: simulated annealing (200-400 ns);<br>3: conventional MD (400-1000 ns) |
| **HT2** | 108 POPE + 36 POPG | 7232 | 32 NA + ions | |
| **RI-HT2** | 108 POPE + 36 POPG | 7897 | 32 NA + ions | |
| **SHf** | 108 POPE + 36 POPG | 9803 | 34 NA + ions | Umbrella sampling, 200 ns each window. |
| **HT2** | 108 POPE + 36 POPG | 10136 | 32 NA + ions | |
| **RI-HT2** | 108 POPE + 36 POPG | 11684 | 32 NA + ions | |

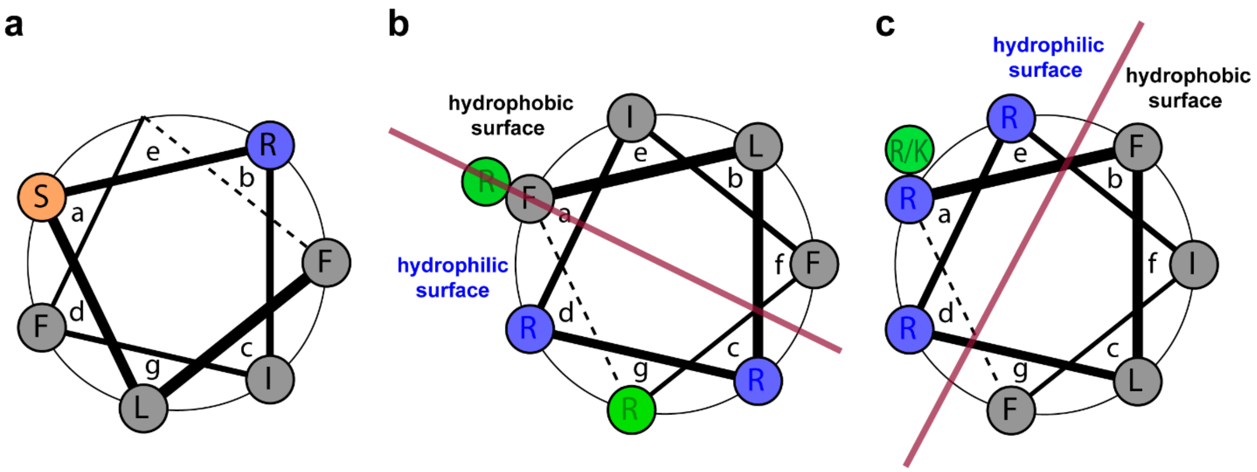


**Figure S1**. **The helical wheel projection of temporin-SHf analogs**. Helical wheel was plotted using the online server DrawCoil 1.0. (**a**) The helical wheel projection of the α-helical structure of temporin-SHf. (**b**) The helical wheel projection of the analogs with head modification. One or two basic residues (green circle) adding to the C-terminus is separated on the hydrophilic surface of the helical cylinder, thereby resulting in increasing amphipathicity of the α-helical. (**c**) The helical wheel projection of the analogs in a tail-to-head fashion. One basic residue Arg or Lys (green circle) added in the middle of the sequence between the α-helical head and hydrophobic aromatic tail is positioned on the hydrophilic surface of the helical cylinder.

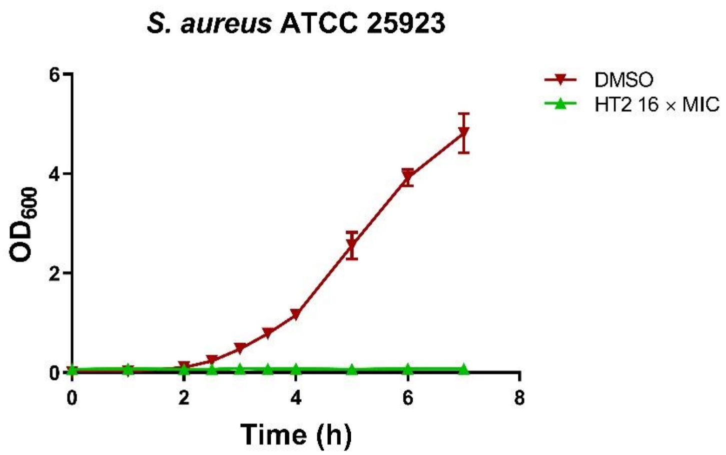


**Figure S2. Growth curves of *S. aureus* ATCC 25923 at 16 × MIC of HT2.**

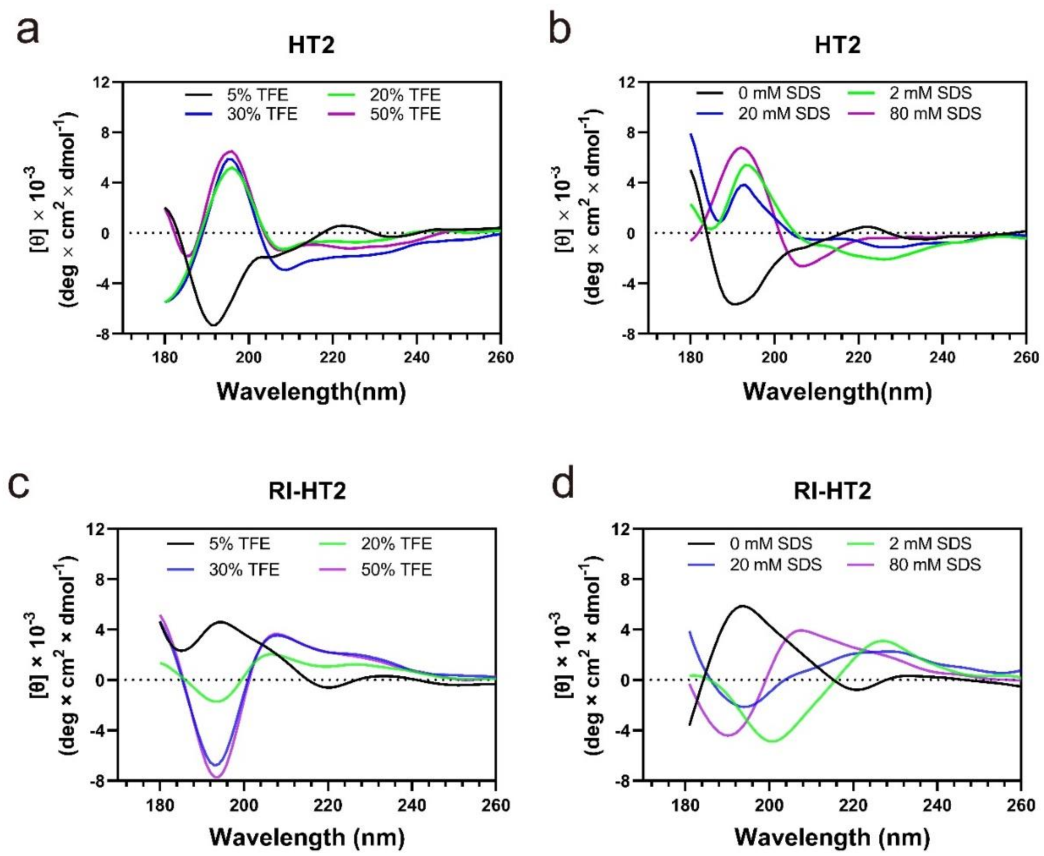


**Figure S3. CD spectra of HT2 and RI-HT2 in presence of membrane mimics.** (**a**) CD spectra of HT2 in presence of trifluoroethanol (TFE). (**b**) CD spectra of HT2 in presence of sodium dodecyl sulfonate (SDS). (**c**) CD spectra of HT2 in presence of TFE. (**d**) CD spectra of HT2 in presence of SDS. The mean residual ellipticity was plotted against wavelength. The values from three scans were averaged per sample, and the peptide concentrations were fixed at 100 μM.

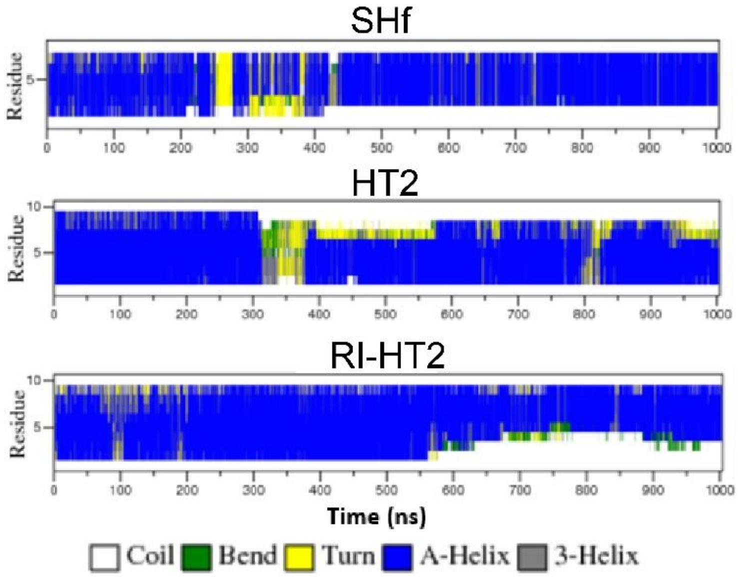


**Figure S4. Secondary structure evolution of SHf, HT2 and RI-HT2 on bacterial membrane.** From 0-200 ns: simulated annealing and elastic network; from 200-400 ns: simulated annealing and from 400-1000 ns: conventional MD.
