# Supplementary material for: Design and evaluation of tadpole-like conformational antimicrobial peptides
Source: Commun Biol. 2023 Nov 18;6:1177. doi: 10.1038/s42003-023-05560-0 (PMC10657444; doi:10.1038/s42003-023-05560-0)
Supplement: Supplementary file 2 — Description of Additional Supplementary Files [file 42003_2023_5560_MOESM2_ESM.docx]

**Description of Additional Supplementary Files**

**File name:** Supplementary Data 1

**Description:** The original numerical data for the graphs
