# Supplementary material for: Design and evaluation of tadpole-like conformational antimicrobial peptides
Source: Commun Biol. 2023 Nov 18;6:1177. doi: 10.1038/s42003-023-05560-0 (PMC10657444; doi:10.1038/s42003-023-05560-0)
Supplement: Supplementary file 4 — Reporting Summary [file 42003_2023_5560_MOESM4_ESM.pdf]

nature portfolio

Corresponding author(s): Aimin Yang

Last updated by author(s): Oct 26, 2023

Reporting Summary

Nature Portfolio wishes to improve the reproducibility of the work that we publish. This form provides structure for consistency and transparency in reporting. For further information on Nature Portfolio policies, see our Editorial Policies and the Editorial Policy Checklist.

Statistics

For all statistical analyses, confirm that the following items are present in the figure legend, table legend, main text, or Methods section.

n/a	Confirmed	
☐	☒	The exact sample size (n) for each experimental group/condition, given as a discrete number and unit of measurement
☐	☒	A statement on whether measurements were taken from distinct samples or whether the same sample was measured repeatedly
☐	☒	The statistical test(s) used AND whether they are one- or two-sided<br>*Only common tests should be described solely by name; describe more complex techniques in the Methods section.*
☐	☒	A description of all covariates tested
☐	☒	A description of any assumptions or corrections, such as tests of normality and adjustment for multiple comparisons
☐	☒	A full description of the statistical parameters including central tendency (e.g. means) or other basic estimates (e.g. regression coefficient) AND variation (e.g. standard deviation) or associated estimates of uncertainty (e.g. confidence intervals)
☐	☒	For null hypothesis testing, the test statistic (e.g. F, t, r) with confidence intervals, effect sizes, degrees of freedom and P value noted<br>*Give P values as exact values whenever suitable.*
☒	☐	For Bayesian analysis, information on the choice of priors and Markov chain Monte Carlo settings
☒	☐	For hierarchical and complex designs, identification of the appropriate level for tests and full reporting of outcomes
☐	☒	Estimates of effect sizes (e.g. Cohen's d, Pearson's r), indicating how they were calculated

Our web collection on statistics for biologists contains articles on many of the points above.

Software and code

Policy information about availability of computer code

Data collection	HPLC, Microplate Readers (Infinite M200 Pro), Field Emission Scanning Electron Microscopy (FEI Sirion200), Transmission Electron Microscopy (Hitachi H-7650, Japan), Applied Photophysics spectrometer (CHIRASCAN), Bruker Avance NMR spectrometers were used to acquire images and datas.
Data analysis	Graphpad prism 8, Adobe Illustrator CS6 overlay method were used to contrast and overlay images as described in the Methods section.<br>The secondary structure of AMP was processed using PyMOL software.<br>All AMP simulations were carried out using GROMACS 2021 as described in the Methods section.<br>All NMR spectra were processed using NMRPipe and analyzed using NMRFAM-Sparky as described in the Methods section. The restraints for backbone torsion angles were derived from chemical shifts using the TALOS software program. The solution structure was calculated with CYANA 2.1 using standard parameters. Structural statistics and global structure quality factors were computed using PSVS version 1.5.

For manuscripts utilizing custom algorithms or software that are central to the research but not yet described in published literature, software must be made available to editors and reviewers. We strongly encourage code deposition in a community repository (e.g. GitHub). See the Nature Portfolio guidelines for submitting code & software for further information.

Data

Policy information about availability of data

All manuscripts must include a data availability statement. This statement should provide the following information, where applicable:

- Accession codes, unique identifiers, or web links for publicly available datasets
- A description of any restrictions on data availability
- For clinical datasets or third party data, please ensure that the statement adheres to our policy

All raw and processed data will be made available upon request.

Research involving human participants, their data, or biological material

Policy information about studies with human participants or human data. See also policy information about sex, gender (identity/presentation), and sexual orientation and race, ethnicity and racism.

Reporting on sex and gender	Not applicable because the study does not involve human research participants.
Reporting on race, ethnicity, or other socially relevant groupings	Not applicable because the study does not involve human research participants.
Population characteristics	Not applicable because the study does not involve human research participants.
Recruitment	Not applicable because the study does not involve human research participants.
Ethics oversight	Not applicable because the study does not involve human research participants.

Note that full information on the approval of the study protocol must also be provided in the manuscript.

Field-specific reporting

Please select the one below that is the best fit for your research. If you are not sure, read the appropriate sections before making your selection.

☒ Life sciences ☐ Behavioural & social sciences ☐ Ecological, evolutionary & environmental sciences

For a reference copy of the document with all sections, see nature.com/documents/nr-reporting-summary-flat.pdf

Life sciences study design

All studies must disclose on these points even when the disclosure is negative.

Sample size	For hemolysis assays, membrane depolarization assay and membrane permeability assay, n=3 were chosen as the minimal replicate number. For antimicrobial activity assay, n≥6 was chosen as the minimal replicate number. For MTT assay, n=6 was chosen as the minimal replicate number. We determined this to be sufficient owing to the low observed variability between each independent experiment.
Data exclusions	Data were not excluded from the analysis.
Replication	All replication attempts were successful and observed positive and negative controls were consistent with previously known results. For the data in this study, we find that each experimental data was consistent with other results.
Randomization	All bacterial colony counts, SEM and TEM for imaging were selected randomly. However, all bacteria that passed quality control were equally analyzed, so there was no requirement for randomization.
Blinding	Blinding was not possible as experimental conditions were evident from the image data.

Reporting for specific materials, systems and methods

We require information from authors about some types of materials, experimental systems and methods used in many studies. Here, indicate whether each material, system or method listed is relevant to your study. If you are not sure if a list item applies to your research, read the appropriate section before selecting a response.

Materials & experimental systems

n/a	Involved in the study	
☒	☐	Antibodies
☐	☒	Eukaryotic cell lines
☒	☐	Palaeontology and archaeology
☒	☐	Animals and other organisms
☒	☐	Clinical data
☒	☐	Dual use research of concern
☒	☐	Plants

Methods

n/a	Involved in the study	
☒	☐	ChIP-seq
☒	☐	Flow cytometry
☒	☐	MRI-based neuroimaging

Eukaryotic cell lines

Policy information about cell lines and Sex and Gender in Research

Cell line source(s)	HEK293T cells (human, ATCC CRL-1573)
Authentication	None of the cell lines have been authenticated.
Mycoplasma contamination	Cell lines were not tested for mycoplasma contamination but no indication of contamination was observed.
Commonly misidentified lines (See ICLAC register)	No commonly misidentified cell lines were used.
